# Recent Advances in Natural Polymers‐based Hydrogels for Periodontal Regeneration

**DOI:** 10.1002/mabi.70194

**Published:** 2026-05-15

**Authors:** João de Freitas Gomes Neto, Tainara de Paula de Lima Lima, Luiza Meurer Brand, Caio Fernando Cardoso Souza, Marcelo Lazzaron Lamers, Pedro L. Granja, Luiz Antonio Pessan, Eduardo Henrique Backes

**Affiliations:** ^1^ Graduate Program in Materials Science and Engineering Federal University of São Carlos São Carlos SP Brazil; ^2^ Department of Morphological Sciences Institute of Basic Health Sciences Federal University of Rio Grande Do Sul Porto Alegre RS Brazil; ^3^ i3S Instituto de Investigação e Inovação Em Saúde Universidade Do Porto Porto Portugal; ^4^ Department of Materials Engineering Federal University of São Carlos (UFSCar) São Carlos SP Brazil

**Keywords:** alginate, chitosan, collagen, hydrogels, natural polymers, periodontal regeneration

## Abstract

Conventional therapies still provide only limited true tissue regeneration for periodontal diseases, particularly periodontitis, which are highly prevalent chronic inflammatory conditions associated with irreversible loss of tooth‐supporting tissues and relevant systemic consequences. In this context, hydrogels based on natural polymers have been widely explored in periodontal tissue engineering as biomimetic matrices capable of modulating the inflammatory response, enabling localized delivery of bioactive agents, and offering temporary structural support. This review summarizes recent advances in hydrogels composed of alginate, collagen, chitosan, and other natural or semi‐synthetic polymers applied to periodontal regeneration. The influence of polymer origin, crosslinking strategies, physicochemical and rheological properties, and processing approaches, including injectable formulations, self‐healing systems, and bioinks for three‐dimensional bioprinting, is discussed in relation to cell adhesion, angiogenesis, osteogenesis, and functional restoration of periodontal tissues. Hybrid platforms such as interpenetrating polymer networks, ceramic‐reinforced composites, and systems designed for controlled delivery of drugs, growth factors, exosomes, and stem cells are also examined, with emphasis on immunomodulatory and stimuli‐responsive designs tailored to the periodontal microenvironment. Despite robust preclinical evidence demonstrating coordinated regeneration of cementum, periodontal ligament, and alveolar bone, major challenges remain. These include the scarcity of well‐controlled clinical trials, limitations in standardization, and regulatory barriers to translation.

## Graphical abstract

Natural polymer‐based hydrogels are emerging as promising biomimetic platforms for periodontal regeneration. This review highlights advances in alginate, collagen, and chitosan hydrogels engineered to modulate inflammation, deliver bioactive agents, and support tissue repair. Hybrid and stimuli‐responsive systems show strong preclinical potential, although clinical translation remains limited by regulatory and standardization challenges.

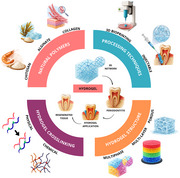

## 1 Introduction

Periodontal diseases are among the most prevalent chronic inflammatory conditions worldwide. Periodontitis, in particular, can lead to irreversible loss of the supporting structures of the teeth, including the gingiva, alveolar bone, periodontal ligament, and cementum. Without intervention, the disease often results in tooth loss and worsens systemic health conditions such as diabetes and cardiovascular disease, thereby imposing a significant public health burden [1, 2]. Conventional treatments such as scaling and root planning can halt disease progression but generally fall short of regenerating lost tissue, highlighting the need for new therapeutic approaches [3].

To address this gap, tissue engineering techniques have emerged as a promising approach, employing scaffolds, bioactive substances, and stem cells to promote the repair of damaged periodontal tissues [4, 5]. In this context, hydrogels derived from natural polymers such as alginate, collagen, gelatin, and chitosan have gained prominence for their biocompatibility, biodegradability, and ability to mimic the extracellular matrix (ECM) environment. These materials act as three‐dimensional (3D) supports, maintaining adequate space for the selective growth of the periodontal ligament and alveolar bone, while also preventing the apical migration of the gingival epithelium [6, 7]. It is important to highlight that natural polymer‐based hydrogels exhibit unique biological activities, including anti‐inflammatory and antimicrobial properties, as well as favorable mechanical and rheological features, which can be tailored for specific regenerative applications [8].

**FIGURE 1:**
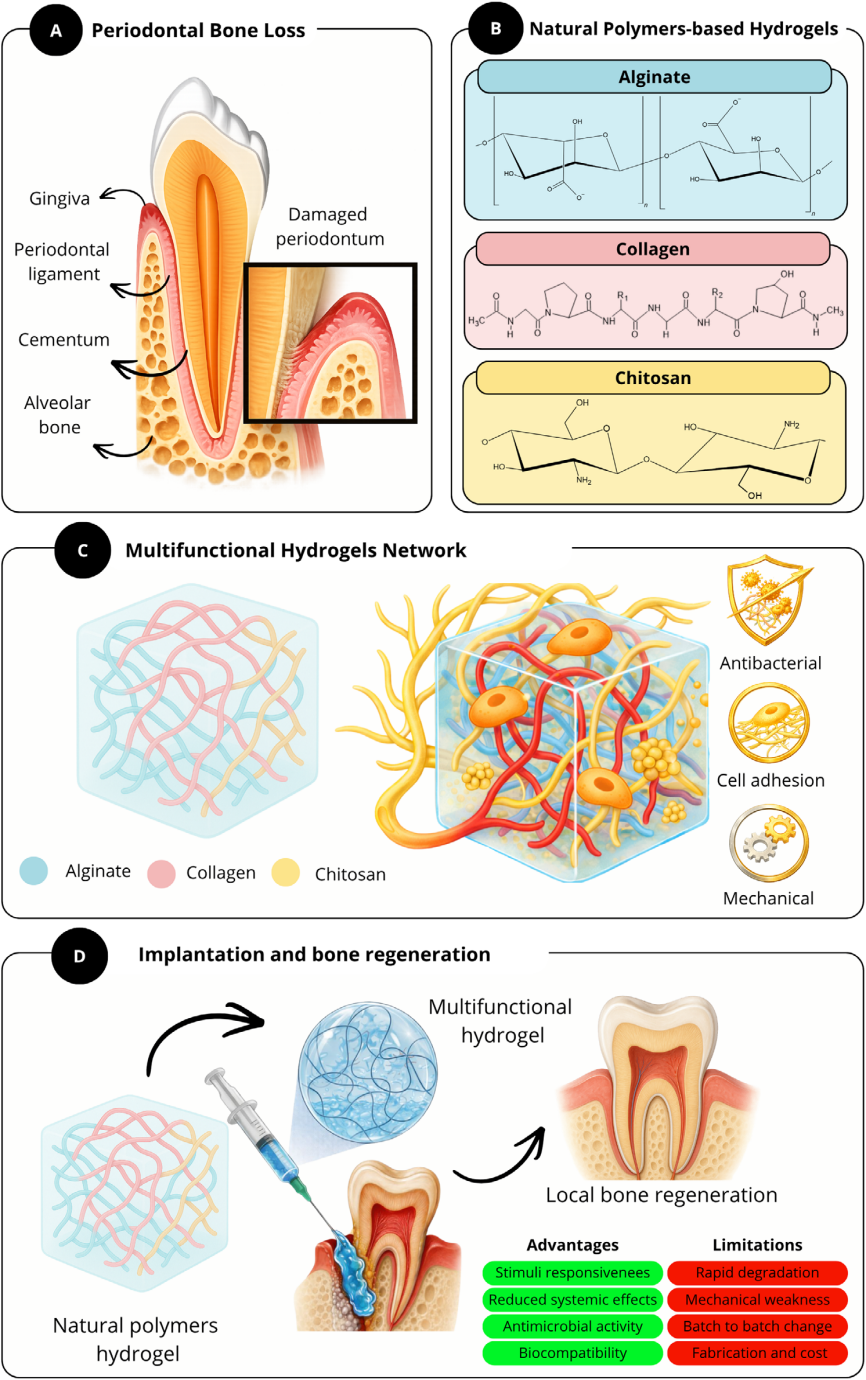
Schematic overview of periodontal bone loss and the rationale for natural polymer‐based hydrogels as a regenerative strategy. (A) Periodontal bone loss resulting from inflammatory periodontal disease, illustrating the main anatomical components of the periodontium, including gingiva, periodontal ligament, cementum, and alveolar bone, as well as the structural damage associated with disease progression. (B) Representative natural polymers commonly used for hydrogel formulation in periodontal tissue engineering—alginate, collagen, and chitosan—together with their characteristic chemical structures. (C) Formation of a multifunctional hydrogel network combining these polymers to generate a three‐dimensional matrix capable of supporting cellular interactions and providing antibacterial, cell‐adhesive, and mechanical functionalities. (D) Local implantation of the injectable hydrogel into periodontal defects promotes tissue repair and bone regeneration. The figure also highlights the main advantages of natural polymer‐based hydrogels, including stimuli responsiveness, reduced systemic effects, antimicrobial activity, and biocompatibility, as well as key limitations such as rapid degradation, limited mechanical strength, batch‐to‐batch variability, and fabrication cost. Created using Canva Pro with AI‐assisted elements from Gemini.

The development of periodontal bone loss and the justification for using hydrogels based on natural polymers as a regenerative approach are comprehensively depicted in Figure 1. It emphasizes how biofilm formation contributes to gingival inflammation and the subsequent bone resorption. The primary biopolymers utilized in hydrogel formulations (alginate, collagen, and chitosan) are then discussed, with a focus on their unique contributions. These include enhanced cell adhesion and ECM biomimicry (collagen), tunable mechanical properties and biodegradation (alginate), and antibacterial activity (chitosan). Last, the figure shows how these functions come together to support biomaterial implantation and encourage local bone regeneration by showing the creation of a combined multifunctional network and its local application within the periodontal defect [9].

Alginate has been widely applied in alveolar bone regeneration due to its adaptability and ability to undergo ionic crosslinking gelation [10, 11]. This allows the creation of hydrogels with mechanical stability and adjustable degradation rates. Its inherent biocompatibility and ease of chemical modification have enabled the incorporation of signaling molecules and nanoparticles, thereby enhancing osteoconductivity and angiogenesis in periodontal defects [12, 13]. Furthermore, hybrid systems that combine alginate with other natural polymers or bioactive ceramics have shown the potential to improve regenerative outcomes. They integrate the favorable biological properties of polysaccharides with the structural robustness of inorganic phases [14, 15]. These composite hydrogels have shown promise in stimulating both soft and hard tissue regeneration, thereby addressing the complexity of the periodontal apparatus.

Collagen and gelatin have proven to be crucial in periodontal tissue engineering strategies. Given their structural similarity to the native ECM, these polymers provide an ideal substrate for cell adhesion, proliferation, and differentiation. Studies have shown that gelatin‐based scaffolds exhibit tunable mechanical properties, favorable degradation rates, and the ability to incorporate bioactive molecules, thereby enabling effective regeneration of craniofacial and periodontal tissues [16, 17]. Similarly, collagen hydrogels have been successfully combined with decellularized matrices, stem cells, and growth factors, enhancing osteogenic and cementogenic differentiation and supporting the formation of functional periodontal ligament fibers in preclinical models [18, 19]. The ability of these scaffolds to replicate an artificial niche capable of stimulating endogenous stem cells represents a shift away from conventional exogenous cell transplantation approaches.

Chitosan, on the other hand, stands out as one of the most extensively studied materials due to its antimicrobial activity, mucoadhesive capacity, and versatility for processing into films, gels, and nanostructures [20, 21]. Chitosan‐based drug delivery systems have been widely investigated for the local delivery of antibiotics and growth factors, enabling controlled release and targeted therapeutic effects in periodontal defects [22]. Moreover, enzymatically crosslinked chitosan hydrogels have shown excellent in vivo biocompatibility and biodegradability, while significantly enhancing functional ligament regeneration even without exogenous cell addition [23]. These findings indicate that chitosan‐based hydrogels not only act as passive space supports but also actively contribute to periodontal regeneration through biological and pharmacological functionalities.

Taken together, recent advances highlight the versatility and clinical promise of natural polymer‐based hydrogels for periodontal regeneration. Although each polymer has its strengths, a common challenge is finding the optimal balance among degradation rate, structural support, and biological activity. New approaches include multifunctional hydrogels capable of sequential drug release, responsiveness to the local inflammatory environment, and guidance of tissue‐specific reconstruction, as demonstrated by convertible hydrogel systems applied in diabetic periodontitis models [24]. Overall, current evidence indicates that natural polymer‐based hydrogels are not merely passive supports but dynamic, bioactive platforms that can orchestrate complex regenerative processes, representing an innovative option for the treatment of periodontitis and associated tissue loss [25, 26, 27].

Recent review studies have highlighted the rapid evolution of hydrogel‐based platforms for periodontal tissue engineering, emphasizing their potential as multifunctional biomaterials capable of combining structural support with localized delivery of therapeutic agents [28, 29]. Advances in injectable and stimuli‐responsive hydrogels, as well as hybrid polymeric systems designed for controlled drug release and immunomodulation, have expanded the therapeutic possibilities for periodontal regeneration [30]. In addition, emerging fabrication approaches, including biofabrication and 3D bioprinting, have enabled improved spatial control over scaffold architecture and cellular organization within periodontal defects [31]. These developments reinforce the growing interest in hydrogel‐based biomaterials as dynamic platforms capable of orchestrating complex regenerative processes in the periodontal microenvironment [32].

This review provides a brief summary of key developments in treatments and techniques for periodontal repair and regeneration using hydrogels derived from natural polymers. By emphasizing the biological benefits, design approaches, and translational possibilities of these biomaterials, we offer a comprehensive view of their present significance and prospects in periodontal treatment. This summary highlights the importance of hydrogels derived from natural polymers in facilitating tissue regeneration and encourages additional interdisciplinary studies.

## 2 Fundamentals of Periodontal Regeneration

The periodontium is a complex tissue composed of four main tissues: root cementum, periodontal ligament, alveolar bone, and gingiva. These tissues, acting in an integrated manner, ensure the support and fixation of the tooth to the alveolar bone while also providing protective functions against the oral microbiota [33].

**FIGURE 2:**
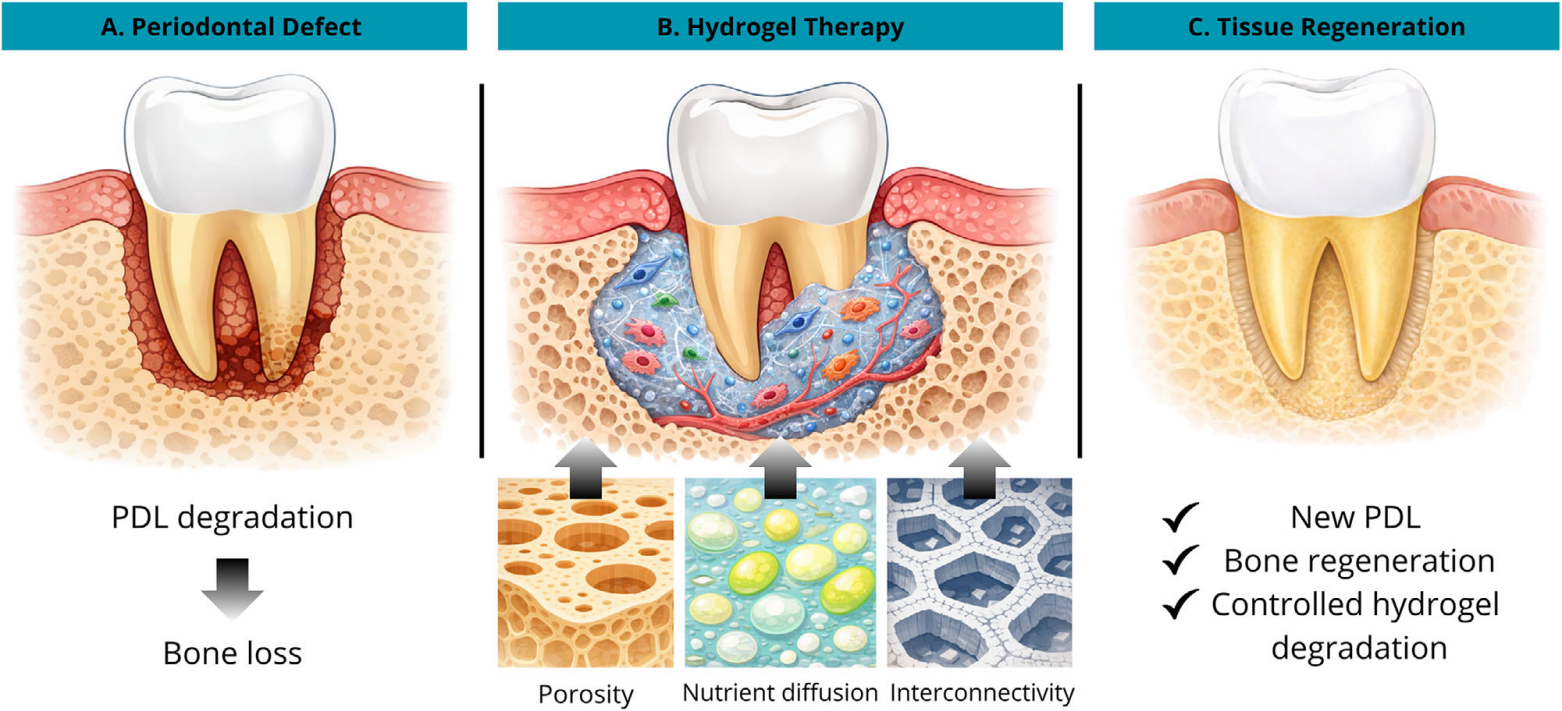
Schematic illustration of hydrogel‐mediated periodontal regeneration. (A) Periodontal defect characterized by degradation of the periodontal ligament (PDL) and consequent alveolar bone loss. (B) Application of a bioactive hydrogel within the defect site provides a supportive microenvironment for cell infiltration, vascularization, and tissue formation. The hydrogel scaffold exhibits key structural features, including porosity, nutrient diffusion capacity, and pore interconnectivity, which facilitate cell migration and metabolic exchange. (C) Regeneration of periodontal tissues, resulting in the formation of a new periodontal ligament, restoration of alveolar bone, and controlled degradation of the hydrogel scaffold during the healing process. Created using Canva Pro with AI‐assisted elements from Gemini.

Periodontal disease, a chronic inflammatory condition affecting over 1 billion people worldwide, is characterized by loss of clinical attachment, gingival bleeding, gingival recession, and periodontal pocket formation (Figure 2A) [34]. If untreated, it can lead to tooth mobility and loss, resulting in functional, aesthetic, and nutritional impairments that reduce quality of life [35]. These sequelae are irreversible, emphasizing the need for therapeutic strategies that go beyond symptom control and aim to restore lost periodontal structures. Severe periodontitis affects 5.8%–49.7% of adults and is a modifiable risk factor for several chronic noncommunicable diseases [36]. Its progression is influenced by disparities in access to oral healthcare, highlighting the need for integrated public health strategies [37, 38].

Successful periodontal regeneration relies on fundamental biological requirements involving both local conditions of the recipient site and the properties of the biomaterial applied (Figure 2B). Critical factors include: (i) effective decontamination of the site and the establishment of a stable blood clot, which serves as a provisional matrix for cellular migration; (ii) maintenance of sufficient space to allow new tissue formation, thereby preventing the undesirable invasion of epithelial and connective tissue cells; and (iii) primary wound closure achieved by proper flap adaptation, which minimizes the risk of bacterial contamination, clot displacement, and healing failure [39].

Periodontal regeneration requires the coordinated participation of multiple cell types, including periodontal ligament fibroblasts, cementoblasts, osteoblasts, and endothelial cells, which adhesion, migration, proliferation, and differentiation are regulated by both molecular signals and the physicochemical features of the microenvironment (Figure 2C) [40, 41]. Growth factors such as platelet‐derived growth factor (PDGF), transforming growth factor beta (TGF‐β), and fibroblast growth factor (FGF) play pivotal roles in this process, promoting angiogenesis, ECM deposition, and mineralization. In parallel, controlling the inflammatory response is critical, as excessive inflammation impairs new tissue formation and promotes fibrosis rather than regeneration [42, 43].

Biomaterials designed for periodontal regeneration should replicate key features of the natural microenvironment, providing structural and biochemical support for tissue development. Parameters such as porosity and pore interconnectivity are critical, as they regulate nutrient diffusion, waste removal, and vascularization [44]. Surface topography also plays a key role, since surface roughness and organization modulate cellular behavior, influencing adhesion and differentiation [44, 45]. For instance, a recent study highlighted that multilayer hydrogels with multidirectional fibers enhanced cell anchorage and promoted their differentiation [46]. Additionally, the biomaterial must exhibit biocompatibility and a degradation rate compatible with new tissue formation, ensuring temporary structural support without inducing adverse inflammatory responses [47].

Multifunctional hydrogels have emerged as promising platforms capable of conforming to periodontal defects and supporting the regeneration of multiple tissues, including alveolar bone, periodontal ligament, cementum, and gingiva as illustrated on Figure 1. Therefore, the ideal biomaterial should combine adequate physiological properties, biocompatibility, controlled degradation profile, and potential for biofunctionalization, thus acting as a biomimetic substrate that promotes cell adhesion, proliferation, differentiation, and migration, while establishing favorable conditions for periodontal regeneration.

## 3 Hydrogels as Biomaterials for Periodontal Regeneration

Hydrogels are soft, semi‐solid materials that feature a 3D polymeric network capable of absorbing large amounts of fluids without disintegrating [48]. This absorption capacity is related to properties such as the presence of hydrophilic functional groups in the polymer chain (─OH, ─CONH_2_, ─NH_2_, ─COOH, ─SO_3_H, and ─CONH─), which allow this structure to (bio)mimic the ECM of tissues [49]. They possess interesting mechanical properties, such as an elastic modulus with values similar to those of native periodontal tissues when crosslinked (10–50 MPa), and a porous architecture (with pore diameters ranging from 20 to 200 µm), creating a favorable environment for the adhesion and proliferation of periodontal cells [50].

Depending on the nature of the bonds that stabilize the polymeric network, hydrogels can be classified as physically or chemically crosslinked systems [51]. In the case of physical crosslinking, these materials form through dynamic non‐covalent interactions rather than chemical ones. Therefore, they tend to be reversible, meaning they are less stable against degradation and have inferior mechanical properties, which can be easily altered by environmental factors such as pH or temperature [52]. Among the types of physical crosslinking, ionic bonding [53], coordination with metal ions [54], hydrophobic interactions [55], and hydrogen bonding stand out [56].

On the other hand, chemical crosslinking involves permanent, stronger interactions between polymer chains via a covalent bond [57]. This type of crosslinking provides greater stability and improved mechanical properties, and is generally achieved via click chemistry [58], Schiff‐base reactions [59], free radical polymerization [60], or oxidation of phenolic groups [61]. In click chemistry, fast and highly selective bond‐forming reactions such as azide–alkyne cycloaddition and thiol–ene coupling, used for functionalization and crosslinking with high yield [61]; in dynamic covalent crosslinking, formation and reversible exchange of covalent bonds (e.g., imines/hydrazones/oximes, boronate–diol complexes, disulfides), enabling network rearrangement and self‐healing [62]; in free radical polymerization, the initiation–propagation–termination sequence of radicals adding to vinyl monomers and, with multifunctional comonomers, producing a crosslinked network [63]; and in phenolic oxidation, such as for instance catechols, bonding occurs when these groups are converted into reactive quinones, which can form crosslinks through C─C/C─O coupling reactions and through reactions with nucleophiles such as amines and thiols via Michael addition and Schiff base formation [61].

The structural complexity of the periodontal region has driven the development of new hydrogel formulations and processing strategies that can promote and accelerate tissue regeneration [64]. Thus, new fabrication techniques have been widely explored, particularly injectable hydrogels and 3D bioprinted scaffolds, which offer greater precision in shaping and functionalizing regenerative matrices. Each of these approaches presents specific characteristics and particular advantages for periodontal repair [65]. Injectable hydrogels, for instance, act directly at the target site, filling periodontal cavities and defects, adapting to the irregular morphology of the tissue, and promoting an environment conducive to regeneration [66]. In contrast, 3D bioprinting combines the biological and mechanical properties of hydrogels with specific cell lineages to develop bioinks that interact with the target tissue [67]. For instance, inkjet bioprinting uses droplet‐based deposition with high cell viability and employs acoustic, piezoelectric, thermal, or hydrodynamic actuation [68]. It offers low cost and simplicity but is limited to low‐viscosity bioinks [69]. These bioinks are printed layer by layer to form 3D scaffolds that directly act on tissue repair, reproducing the cellular microenvironment's conditions and favoring tissue regeneration [70].

Self‐assembling hydrogels form through the spontaneous organization of bioactive components, eliminating the need for external crosslinkers. They exhibit shear‐thinning and self‐healing properties, allowing them to conform to irregular bone defects while preserving mechanical control [71].

Recent studies have demonstrated the potential of both techniques for periodontitis treatment. The in vivo evidence that injectable, thermosensitive hydrogels can combine periodontal tissue repair with localized anti‐inflammatory action is summarized in Figure 2. The formulation allows for defect filling and retention in a rat periodontitis model by remaining injectable at low temperatures and gelling at physiological temperatures (Figure 2B). Its porous microstructure may facilitate transport and cell‐matrix interactions. The potential of these multifunctional hydrogel systems for periodontal regeneration is supported by overall imaging and histological results that showed improved alveolar bone preservation/reconstruction and decreased inflammatory burden (Figure 2C) [13, 72].

In the study of Liu et al., the authors investigated an injectable thermosensitive hydrogel formulated from chitosan, beta‐glycerophosphate, and gelatin as a local delivery platform for interleukin‐1 receptor antagonist to mitigate inflammation in a diabetic periodontitis context [72]. The material showed in vitro biocompatibility (cell viability testing in the murine macrophage cell line RAW 264.7) and a clear anti‐inflammatory profile, evidenced by reduced mRNA expression of interleukin (IL)‐1 beta, IL‐6, and tumor necrosis factor (TNF) alpha under inflammatory stimulation with lipopolysaccharide and high‐glucose conditions, alongside in vivo safety indicators (liver and kidney histology by hematoxylin and eosin staining, and serum creatinine, aspartate aminotransferase, and alanine aminotransferase without significant intergroup differences) and an in vivo anti‐inflammatory effect in rats, where treatment with the IL‐1 receptor antagonist‐loaded chitosan/beta‐glycerophosphate/gelatin hydrogel significantly lowered inflammatory cytokine expression relative to diabetic periodontitis controls; additionally, the study reports that this thermosensitive hydrogel can release IL‐1 receptor antagonist continuously for up to 21 days, supporting sustained local inhibition of inflammation [72].

**FIGURE 3:**
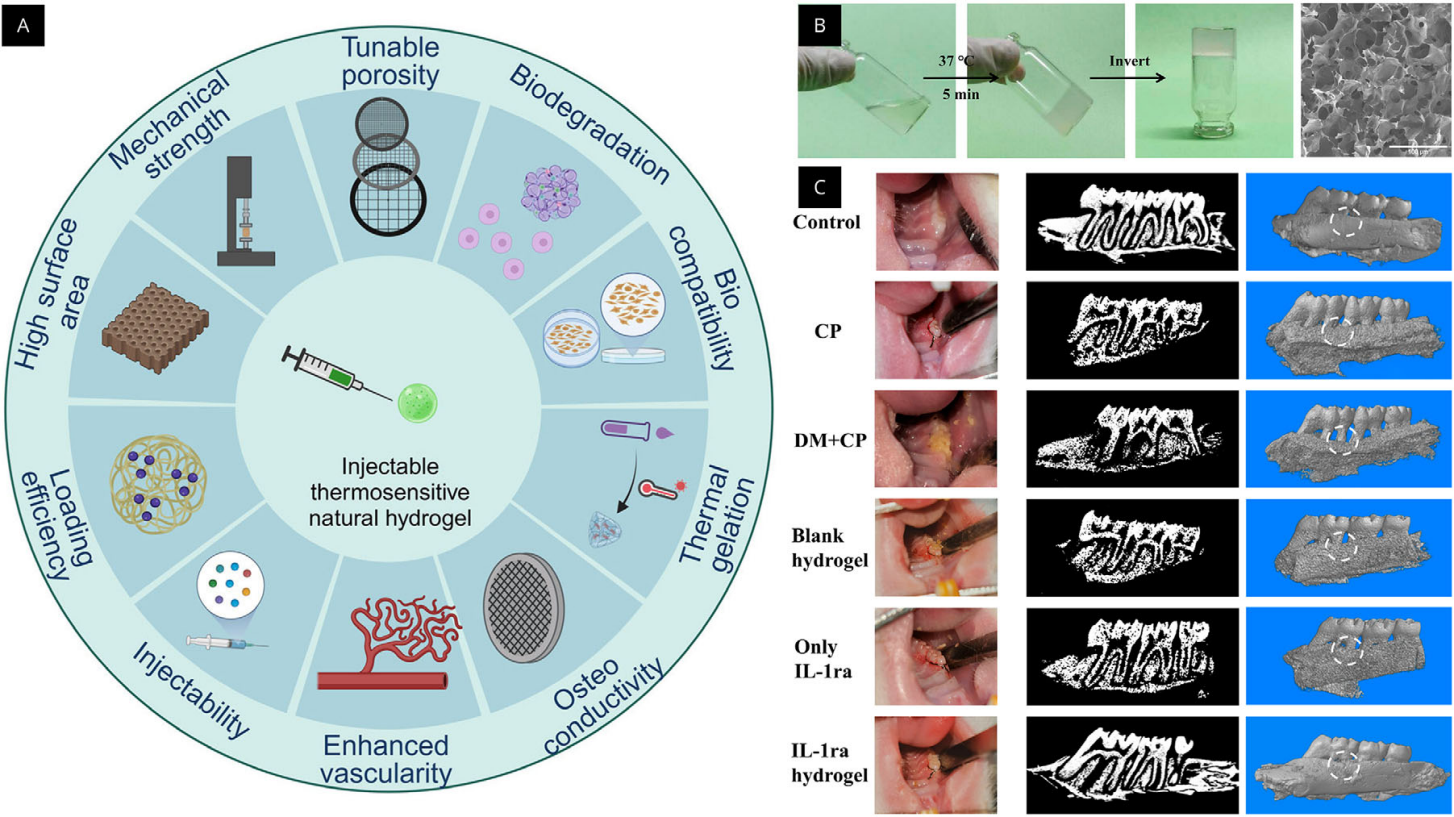
(A) Schematic overview of key performance requirements for an injectable thermosensitive natural hydrogel, including injectability, thermal gelation, biocompatibility, biodegradation, tunable porosity, mechanical strength, osteoconductivity, loading efficiency, and enhanced vascularization. (B) Sol–gel transition at 37°C within 5 min demonstrated by vial inversion, and a representative scanning electron microscopy image (SEM) showing the porous microstructure of the hydrogel. (C) Representative intraoral photographs, binarized micro‐computed tomography (µCT) slices, and 3D reconstructions of alveolar bone for the experimental groups: control, chronic periodontitis, diabetes mellitus + chronic periodontitis, blank hydrogel, free interleukin (IL)‐1 receptor antagonist, and IL‐1 receptor antagonist‐loaded hydrogel (dashed circles indicate the defect/region of interest). Reproduced with permission from the original publisher (Open Access) under the Creative Commons Attribution 4.0 International License (CC BY 4.0): panel A from El‐Nablaway et al. [13]; panels B and C from Liu et al. [72]. https://creativecommons.org/licenses/by/4.0/.

Regarding the 3D bioprinting technique, studies such as that by De Souza Araújo et al. demonstrated the development of bioprinted collagen‐based 3D scaffolds containing periodontal ligament stem cells (PDLSCs) for periodontal regeneration [73]. The study aimed to evaluate cell viability, differentiation, and organization at different collagen concentrations, exploring the potential of bioprinting as a precise and reproducible technique for tissue reconstruction, as shown in Figure 3. Collagen type I bioinks at concentrations of 10 and 15 mg/mL were loaded with PDLSCs and processed by extrusion bioprinting, exhibiting good shear‐thinning behavior and structural fidelity [73]. The samples exhibited high cell viability (greater than 95%), maintained morphology, and osteogenic differentiation under inductive conditions, with expression of markers such as Runt‐related transcription factor 2 (RUNX2), alkaline phosphatase (ALP), osteocalcin (OCN), and collagen type I alpha 1 chain (COL1A1). In vivo tests showed that the bioprinted scaffolds, implanted subcutaneously in mice and applied directly to root surfaces, demonstrated biocompatibility, adhesion to the tooth surface, and the formation of organized connective tissue with cells aligned parallel to the root, particularly in the 15 mg/mL formulations [73].

### 3.1 Alginate‐Based Hydrogels

Alginate is a naturally derived anionic polysaccharide, obtained mainly from brown seaweeds, and is widely employed as a biomaterial due to its biocompatibility, low toxicity, and ability to form hydrogels under mild conditions [74]. Chemically, it consists of blocks of β‐D‐mannuronic acid (M) and α‐*L*‐guluronic acid (G), whose proportion and distribution influence properties such as gel stiffness, porosity, and stability [75, 76]. Gelation generally occurs via ionic crosslinking with divalent cations (especially Ca^2^^+^), forming a 3D network described by the “egg‐box” model, which enables the encapsulation of cells and biomolecules without exposing them to organic solvents or high temperatures [77]. Consequently, alginate hydrogels have been extensively investigated for tissue engineering and controlled‐release systems; however, their limited cell adhesiveness and degradation predominantly driven by ionic exchange have motivated modification strategies to enhance cell–matrix interactions and mechanical performance in regenerative applications [78].

Studies have indicated that periodate‐mediated oxidation of alginate can increase its biodegradation rate and enhance its long‐term biological safety [79, 80]. For example, pioneering studies by Boontheekul et al. showed that alginate gel degradation can be tuned by partial oxidation of the polymer chains and by employing a bimodal molecular weight distribution. Specifically, sodium periodate oxidation introduces hydrolytically cleavable linkages into the polysaccharide backbone and shows that increasing the degree of oxidation accelerated degradation and enables control over the degradation kinetics [81].

Another approach to enhance cell adhesion in alginate hydrogels is to compensate for the lack of intrinsic cell‐binding sites by incorporating the arginine–glycine–aspartic acid (RGD) adhesion ligand [82]. Preclinical evidence supported the biocompatibility and pro‐regenerative performance of RGD‐modified alginate/gelatin‐methacrylate (GelMA) hydrogel sheets as cell‐delivery matrices, demonstrating sustained cell viability and favorable tissue integration [83]. In a murine full‐thickness excisional wound model aimed at soft tissue regeneration, gingiva‐derived mesenchymal stem cells delivered in an alginate‐RGD/GelMA hydrogel sheet improved granulation tissue organization and collagen deposition, reduced TNF‐α and increased IL‐10, and enhanced angiogenesis, with immunostaining confirming human cell engraftment [83].

In parallel to these chemical modifications, formulation approaches, such as blending alginate with other polymers or incorporating functional components, are widely used to tailor hydrogel mechanics, swelling, and transport behavior, and biological signaling [84, 85, 86]. Natural polymers (e.g., gelatin and hyaluronic acid (HA)) can provide biomimetic cues and improve cell–matrix interactions, while synthetic polymers (e.g., poly(lactic‐*co*‐glycolic acid) (PLGA), poly(ethylene glycol) (PEG), or poly(vinyl alcohol) (PVA)‐based networks) may increase mechanical robustness and yield more predictable viscoelastic and diffusion behavior [87]. Additionally, inorganic phases (e.g., hydroxyapatite (HAp), beta‐tricalcium phosphate, and bioactive glass) can be introduced to adjust stiffness and confer application‐specific bioactivity [79].

**FIGURE 4:**
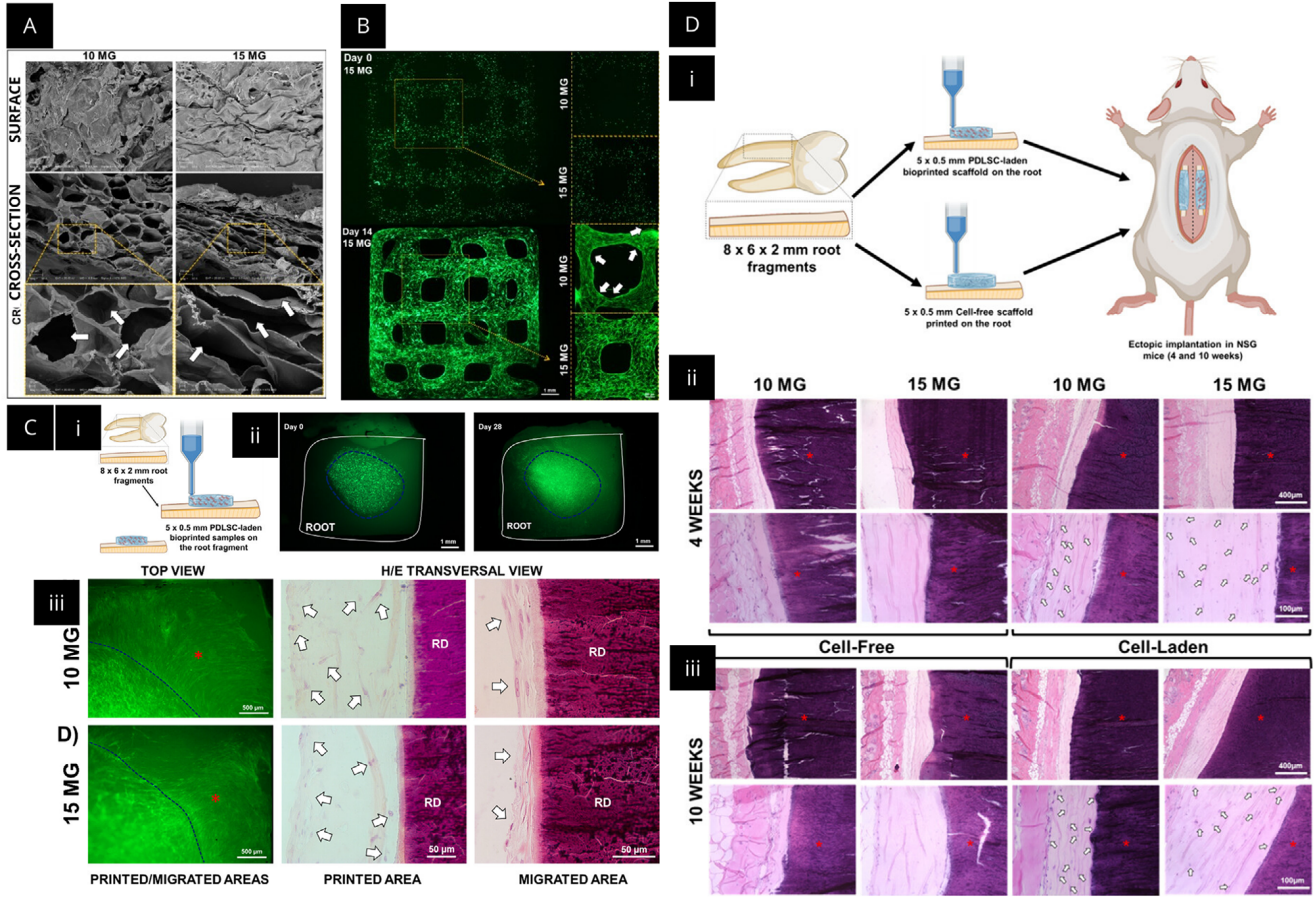
Extrusion‐based 3D bioprinting of collagen hydrogels with periodontal ligament stem cells (PDLSC) for periodontal ligament regeneration. (A) Scanning electron microscopy images of the surface and cross‐sections of constructs prepared at 10 and 15 mg/mL, showing a porous architecture. (B) Fluorescence microscopy of box‐shaped bioprinted scaffolds containing green fluorescent protein‐labeled PDLSC at days 0 and 14, indicating good cell distribution and scaffold remodeling. (C) Schematic of bioprinting PDLSC‐laden constructs on tooth root fragments and representative fluorescence microscopy and histology after culture, showing printed and migrated cell regions on radicular dentin. (D) Hematoxylin and eosin histology at 4 and 10 weeks after ectopic implantation in immunocompromised NOD scid gamma mice, comparing cell‐free versus PDLSC‐laden constructs printed with 10 and 15 mg/mL collagen. Reproduced with permission from American Chemical Society, ACS Applied Materials & Interfaces (de Souza Araujo et al.) [73], licensed under Creative Commons Attribution–NonCommercial–NoDerivatives 4.0 International (CC BY‐NC‐ND 4.0). https://creativecommons.org/licenses/by‐nc‐nd/4.0/.

The versatility of alginate‐based injectable hydrogels has been demonstrated in preclinical periodontitis models through systems engineered for sequential therapeutic delivery [88]. In a rat periodontitis model [89] (Figure 4A), a dopamine‐grafted alginate, hydrogel co‐loaded with methylene blue (MB) and PLGA microspheres encapsulating Semaphorin 3A (Sema3A) enabled in situ application and coordinated antibacterial, anti‐inflammatory, and bone‐regenerative effects. MB was released rapidly to support photodynamic antibacterial therapy, followed by sustained Sema3A release to promote macrophage polarization toward a reparative phenotype and enhance osteogenesis (Figure 4C) [89].

In another study, a bioink composed of GelMA/alginate with bioactive glass microspheres (BGM) was developed for 3D bioprinting of scaffolds aimed at periodontal regeneration [90]. The addition of BGM increased bioactivity (through induction of apatite formation), improved stability and degradation behavior, and enhanced in vitro osteogenesis (higher ALP activity, mineralization, and osteogenic gene expression), while maintaining good biocompatibility. In bioprinted scaffolds loaded with mouse bone marrow‐derived mesenchymal stem cells and growth factors, bone morphogenetic protein (BMP) 2 promoted osteogenic differentiation, whereas PDGF stimulated markers associated with soft tissue repair. In an in vivo Beagle dog model, scaffolds containing cells supplemented with BMP‐2 and platelet‐derived growth factor achieved the greatest regeneration of gingiva, periodontal ligament, and alveolar bone after 8 weeks, supporting the system's potential for periodontal reconstruction [90].

### 3.2 Collagen‐Based Hydrogels

Collagen, mainly type I, is the dominant protein in periodontal connective tissues [91]. It has long been considered the benchmark natural polymer for regenerative strategies, as its fibrillar architecture and biochemical cues closely resemble the native ECM [91]. Recent developments highlight both mammalian and fish‐derived collagen as viable sources, with fish collagen being incorporated into bilayer membranes to achieve hydrophilicity and osteoconductivity, while also providing structural stability when blended with polymers such as PVA [92]. Beyond barrier membranes, collagen has been combined with elastin‐like polypeptides and bioactive glass, generating hydrogels that balance compressive strength and porosity while supporting the osteogenic differentiation of adipose‐derived stem cells [93]. In line with these advances, recent reviews underscore collagen's role not only as a scaffold but also as a versatile delivery system for bioactive molecules and cells [94].

The excellent biocompatibility of collagen hydrogels has been consistently reported across preclinical studies. In a canine model, type I collagen crosslinked with ascorbate–copper ions was shown to integrate into periodontal defects, and when enriched with FGF2, it promoted the formation of cementum‐like tissue and oriented periodontal ligament (PDL) fibers (i.e., collagen fiber bundles of the periodontal ligament that normally insert into cementum and alveolar bone and provide functional tooth support) without ankylosis [95]. Similarly, in a rat fenestration defect model, in situ photo‐crosslinked collagen hydrogels seeded with human PDL fibroblasts reduced epithelial downgrowth and improved bone regeneration [91]. At the scaffold surface, collagen coatings combined with GelMA have also enhanced adhesion, proliferation, and mineralization of osteogenic cells in vitro, confirming collagen's ability to act as a bioactive interface within multilayer constructs [96].

A central attribute of collagen hydrogels lies in their affinity for host cells (meaning their capacity to support cell attachment, spreading, migration, and survival through collagen‐binding interactions) and in acting as depots for bioactive factors. In a rat periodontal defect model, a type I collagen hydrogel loaded with decellularized tooth and periodontal ligament matrix promoted endogenous regeneration of cementum, periodontal ligament, and alveolar bone within 60 days [18, 19]. Other studies have leveraged collagen as a delivery platform for exogenous cells. For example, a composite scaffold integrating PCL, collagen, and cellulose acetate, combined with a collagen hydrogel encapsulating PDLSCs and curcumin‐loaded nanoparticles, significantly reduced pro‐inflammatory cytokines (tumor necrosis factor alpha and interleukin 6) while upregulating regenerative mediators such as TGF‐β and basic FGF [16]. These findings highlight collagen's dual role as both a structural framework and a regulator of the inflammatory microenvironment.

Additionally, collagen‐based hydrogels have been strategically combined with autologous platelet‐rich plasma concentrates. The incorporation of injectable platelet‐rich fibrin into collagen–chitosan hydrogels stabilized the rapid release profile of growth factors, achieving sustained delivery of TGF‐β1 and PDGF AB for up to 17 days [97]. Parallel in vitro and in vivo studies demonstrated that such hybrid systems enhanced ALP activity, mineral deposition, and preservation of alveolar bone volume in extraction socket models [98]. Importantly, Fourier transform infrared (FTIR) spectroscopy confirmed that these synergies were based on physical rather than chemical bonding, thus maintaining the intrinsic properties of both collagen and platelet‐rich fibrin [97].

**FIGURE 5:**
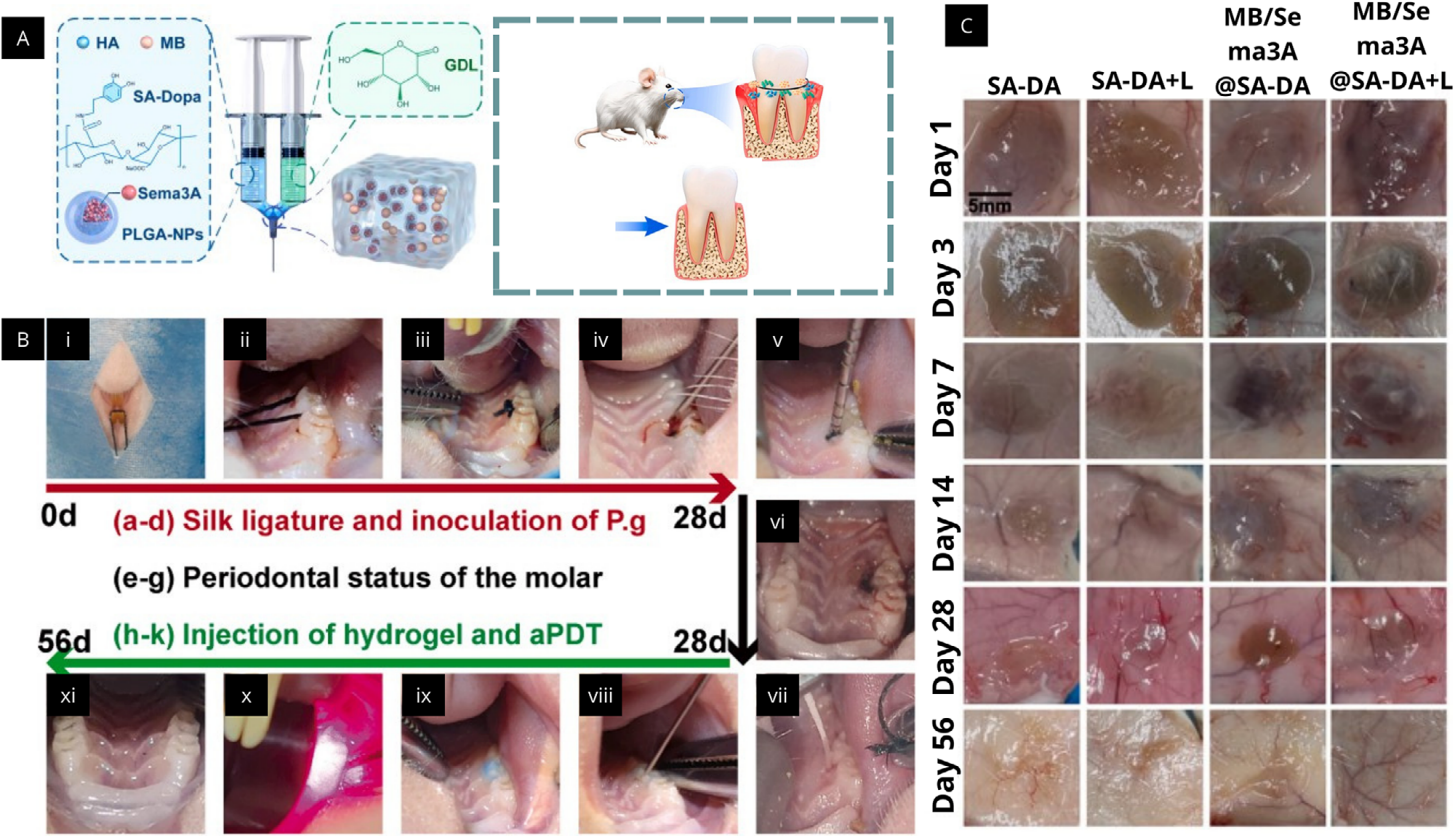
(A) Schematic illustration of the dopamine‐modified alginate (SA–Dopa) hydrogel system incorporating hyaluronic acid (HA), methylene blue (MB), and Semaphorin 3A (Sema3A) delivered via poly(lactic‐co‐glycolic acid) nanoparticles, with gelation mediated by glucono‐δ‐lactone for localized retention and controlled release in periodontal tissues. (B) Experimental workflow of the ligature‐induced periodontitis model in rats: (i–iv) placement of silk ligatures and inoculation with *Porphyromonas gingivalis* (P.g.); (v, vi) evaluation of periodontal status; (vii–xi) injection of the hydrogel followed by antimicrobial photodynamic therapy using 660 nm laser irradiation. (C) Representative macroscopic images of subcutaneous injection sites at different time points (Day 1–56), comparing hydrogel formulations and showing progressive biodegradation and local tissue response. Reproduced with permission from Elsevier: Xu et al. [89], Chemical Engineering Journal. Permission obtained via the publisher's licensing service.

Taken together, collagen‐based hydrogels exhibit remarkable versatility, supporting periodontal regeneration through multiple mechanisms: biocompatibility and tissue integration, cell recruitment and delivery, modulation of inflammatory signaling, and controlled release of growth factors. Across animal models, they have demonstrated true periodontal regeneration characterized by cementum formation, oriented PDL fibers, and alveolar bone reconstruction, as can be seen on Figure 5 [16, 19, 91, 95]. Current evidence positions collagen not only as a cornerstone of natural polymer hydrogels for periodontal therapy but also as a modular platform for multifunctional constructs that synchronize mechanics, degradation, and biological signaling [93, 94, 99].

**FIGURE 6:**
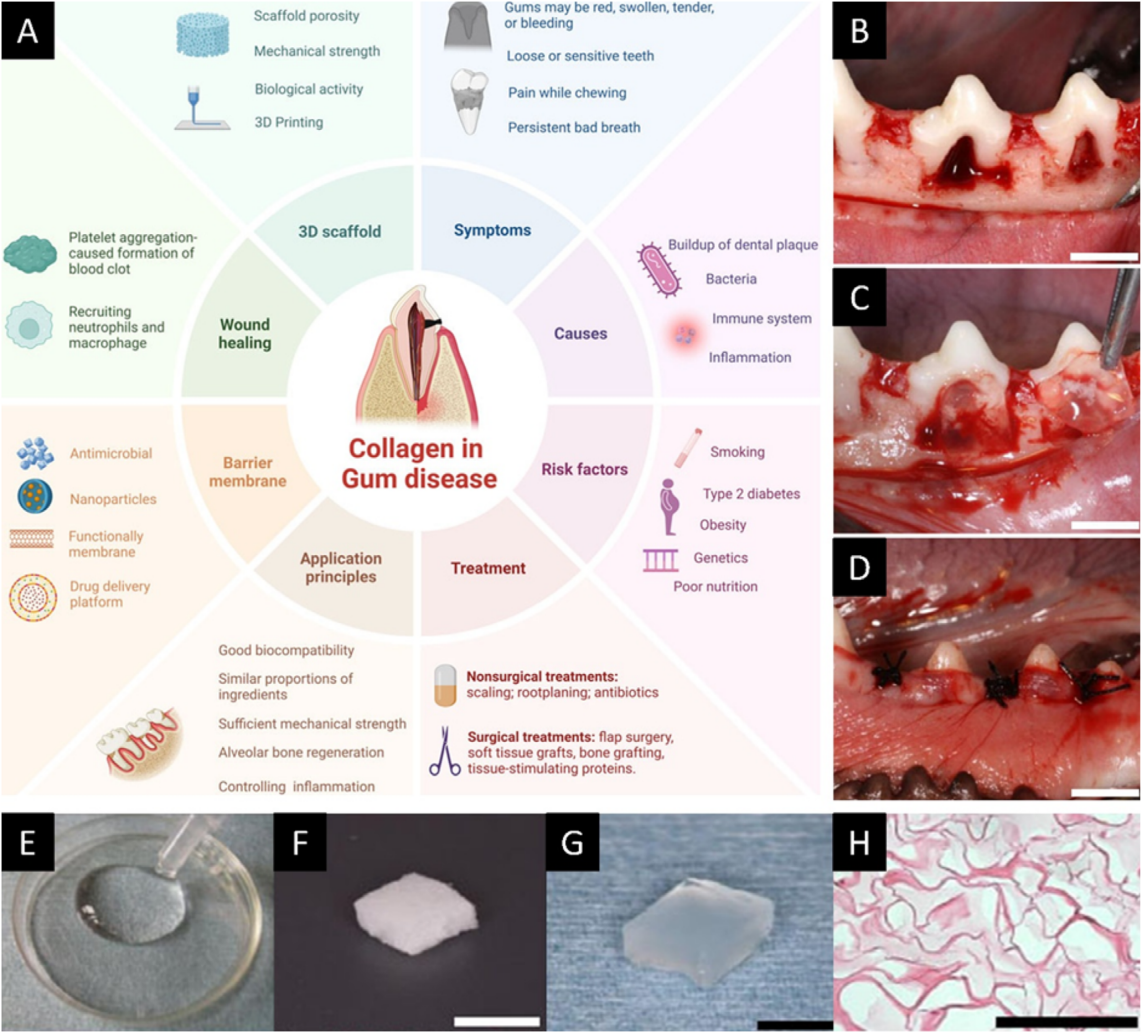
Collagen‐based strategies for periodontal regeneration. (A) Schematic representation of periodontal disease and main collagen‐based therapeutic applications, including treatment principles and tissue engineering approaches. (B–D) Class II furcation defect model (scale bars represent 5 mm): (B) creation of standardized defects; (C) implantation of the collagen scaffold; (D) postoperative appearance after suturing. (E–H) Different collagen biomaterial formats (scale bars represent 5 mm (F,G) and 100 µm (H)): (E) hydrogel; (F) sponge; (G) hydrogel scaffold; (H) histological section showing its porous structure. Reproduced with permission from the original publisher (Open Access) under the Creative Commons Attribution 4.0 International License (CC BY 4.0): panel A from Feng et al. [99], panel B–H from Momose et al. [95]. https://creativecommons.org/licenses/by/4.0/.

Nonetheless, the full clinical potential of collagen‐based hydrogels has yet to be realized. Several limitations remain, including low gelation rates under physiological conditions, limited availability of high‐purity sources, high production costs, and inherently poor mechanical strength compared to synthetic polymers. These drawbacks restrict their stand‐alone use in load‐bearing periodontal defects and necessitate further optimization or combination with reinforcing agents and advanced crosslinking strategies [99]. Figure 6E–G illustrates collagen‐based strategies for periodontal regeneration, highlighting different collagen scaffold formats and their application in a preclinical class II furcation defect model (Figure 6B–D).

### 3.3 Chitosan‐Based Hydrogels

**FIGURE 7:**
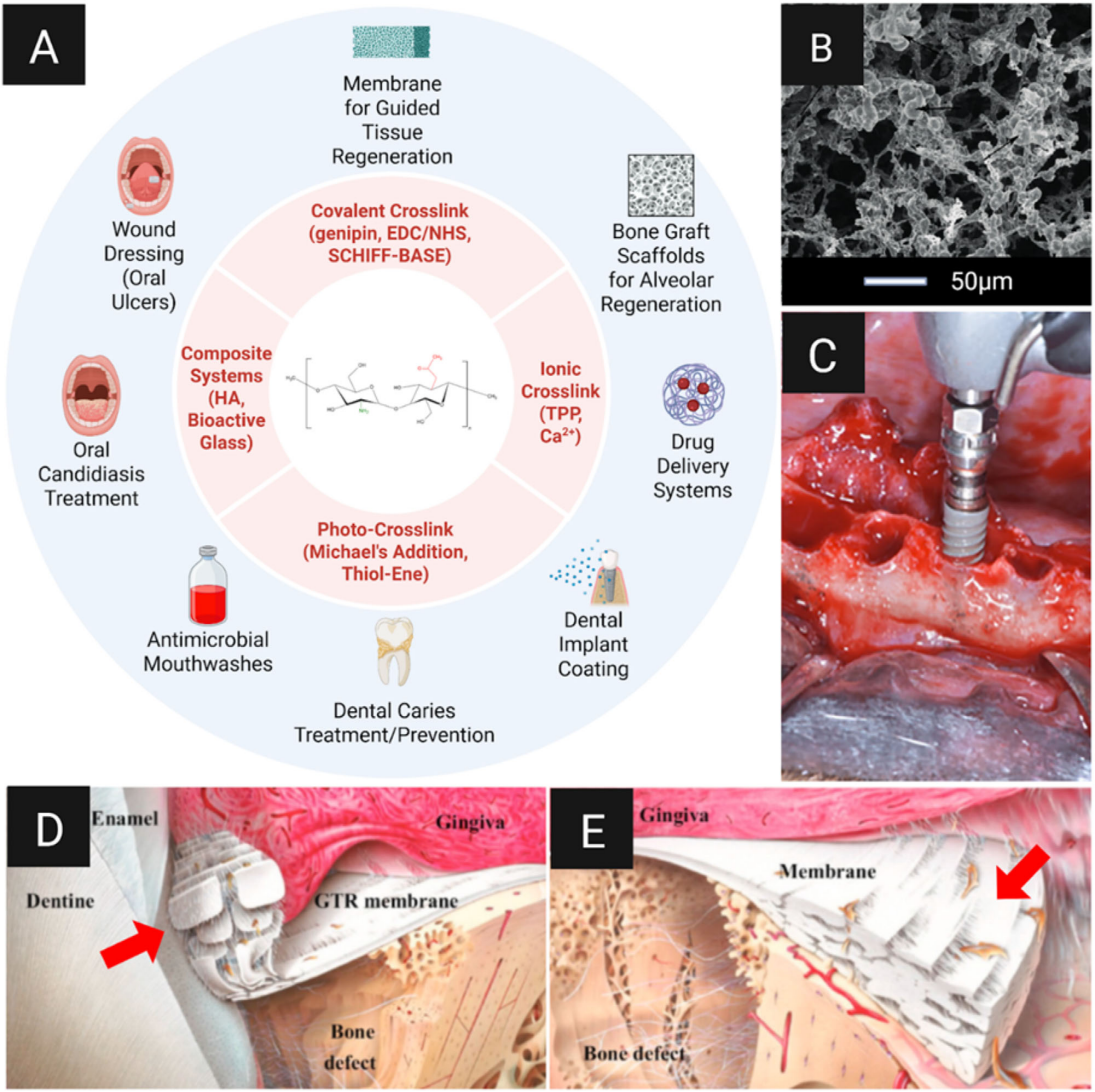
Chitosan biomedical applications for oral treatments. (A) Scheme of general applications and examples of chitosan in oral treatments. (B) Scanning electron microscopy (SEM) image of chitosan hydrogel loaded with stem cells from the apical papilla (scale bar: 50 µm). (C) Insertion of chitosan‐coated titanium implant. (D,E) Scheme of inserted chitosan membrane between the gingiva and bone defect to avoid infection and stimulate tissue regeneration; red arrows: membrane (white). Reproduced with permission from the original publisher (Open Access) under the Creative Commons Attribution 4.0 International License (CC BY 4.0): panel A created using Canva Pro with AI‐assisted elements; panel B from Moreira et al. [108]; panel C from López‐Valverde et al. [109]; panels D,E from Husain et al. [110]. https://creativecommons.org/licenses/by/4.0/.

Chitosan is a natural biopolymer belonging to the polysaccharide category. Its structure is based on β‐(1→4)‐linked D‐glucosamine and N‐acetyl‐*D*‐glucosamine, as shown in Figure 7, and it is produced by the deacetylation of chitin, which is found in the exoskeletons of arthropods such as crustaceans and arachnids [100]. It exhibits a high level of biocompatibility, and its physicochemical behavior is governed by the degree of deacetylation. In its hydrogel form, chitosan can be structured via ionic complexation (e.g., tripolyphosphate and anionic polyelectrolytes), covalent crosslinking (e.g., genipin, 1‐ethyl‐3‐(3‐dimethylaminopropyl)carbodiimide (EDC)/N‐hydroxysuccinimide (NHS), and oxidized polysaccharides via Schiff‐base chemistry), or photo‐crosslinking, as demonstrated in Figure 7A. This makes it possible to create printable and manageable hydrogels [101, 102, 103, 104]. Dual‐responsive thermo‐pH systems can be created by combining polymers, for example, chitosan with beta‐glycerophosphate or chitosan with hyaluronic acid, as well as composite formulations with nanofillers (HAp, bioactive glass, halloysite, and nanosilica), which can produce materials with more controllable properties [105]. Furthermore, they offer mechanical reinforcement, the possibility of three‐dimensional printing, and the sustained release of specific contents under diffusion‐ and erosion‐governed regimens [97, 105, 106, 107].

The use of chitosan in oral applications is mainly related to its cationic charge, mucoadhesion, and antibiofilm activity against oral pathogens [111]. In the case of periodontitis, for example, the cationic surface of chitosan disrupts bacterial membranes and reduces the formation of biofilms (*Porphyromonas gingivalis* and *Streptococcus mutans*) [112, 113]. Chitosan‐based systems demonstrated high versatility and enhanced potential for tissue repair in the recovery from bone fractures or alveolar perforations due to their ability to stimulate osteoblasts [114]. When combined with HAp or PVA, for example, chitosan hydrogels can mimic the ECM in a 3D scaffold environment, thereby facilitating osteoblast adhesion, proliferation, and mineral deposition [103, 115]. Chitosan hydrogels can also be injectable when they present adequate rheological properties, which leads to better manipulation of the material to cover alveolar defects [115]. Meanwhile, oral inflammation activities stimulate angiogenesis and the re‐establishment of the original tissue [116].

Moreover, biomaterials with antimicrobial properties have gained increasing attention in periodontal regeneration, as infection control is a prerequisite for therapeutic success. These antimicrobial effects may be either intrinsic to the hydrogel matrix or achieved through the incorporation of bioactive agents. Intrinsically antimicrobial hydrogels, such as those based on cationic polymers like chitosan, exert their effects through direct interactions with bacterial cell membranes, reducing bacterial adhesion and biofilm formation while minimizing the risk of antimicrobial resistance [117]. Alternatively, hydrogels can be functionalized with antimicrobial agents, including antibiotics, metal ions, nanoparticles, or antimicrobial peptides, enabling localized and sustained release at the defect site. This strategy allows effective bacterial control while limiting systemic exposure and preserving a microenvironment conducive to cell viability and tissue regeneration [117, 118].

The ability to incorporate bioactive agents represents an important advantage. In a study, it was demonstrated that the addition of lyophilized platelet concentrate to a chitosan‐based hydrogel enabled the sustained release of TGF‐β1 and PDGF, resulting in greater viability of encapsulated cells. Similarly, the incorporation of growth factors, such as FGF‐2 and PDGF‐5, enhanced new bone formation in animal models [119]. Another promising modification is the functionalization of hydrogels with arginine–glycine–aspartic acid (RGD) peptides, which improved cell adhesion, spreading, and mineralization [120]. In addition to their regenerative properties, hydrogels with antibacterial activity have been investigated as a complementary strategy, since controlling periodontal infection is a key prerequisite for therapeutic success [121, 122].

In the context of dentistry, chitosan's anti‐microbial properties have been investigated for the management of *Candida albicans* infections and stomatitis lesions, as well as for the treatment of mucosal ulcers and in odontological restoration procedures [123, 124]. Chitosan has been incorporated into hydrogels and adhesive compositions to exploit its antibiotic and regenerative properties, thereby reducing bacterial activity and preventing the formation of secondary caries [123, 124]. Furthermore, its applications also include guided tissue regeneration membranes, bone graft scaffolds, antimicrobial mouthwashes, gels, drug delivery systems, dental implant coatings, wound dressings, endodontic irrigants and sealers, remineralizing agents for enamel, dental adhesives and composites, hemostatic agents, caries prevention, and treatments for oral candidiasis and mucosal infections [125, 126, 127].

Of course, chitosan is not a perfect biomaterial, since the inks for 3D printing and bioprinting face a number of limitations, which include limited printability, weak mechanics, and pH‐dependent solubility [128]. Each crosslinking route only partially overcomes these issues. Ionic crosslinking is a mild method that can produce fragile and reversible networks, while composite systems with other polymers improve strength and viscosity, but at the cost of higher formulation complexity of chemical arrangements and in production terms [128]. Covalent crosslinking and photocrosslinking yield more stable and precise structures, but also require chemical modifications and reactive or photoactive components, which have the potential to compromise cell viability and complicate process control [129, 130].

As an isolated material, chitosan presents certain challenges in terms of its utilization. The material is only soluble under acidic conditions, at a pH below 6, which leads to the necessity to treat the material completely in such conditions before any kind of applications, which is not conducive to the viability of most cells to be imbued or treated in direct contact with the material [131]. To improve the solubility of chitosan, the polymer needs to be modified before any use, as N, O‐carboxymethyil chitosan, N‐acylated chitosan, N‐alkylated chitosan, and other modifications that can be used to prepare neutralized bioinks using chitosan as the base biomaterial [131, 132].

In the event of the combination of polymers, rheological difficulties are the most salient problems. In order to achieve the ideal degree of printability, it is essential that the proportions are very precise, and when the subject is dental applications, it is necessary to determine the concentrations for the enamel, the dentin, and the bone composition of the tooth [110]. It has been demonstrated that this can result in a reduction of porosity, nutrient diffusion, and cell viability at the conclusion of the application process.

As an exemplar, alginate‑carboxymethylchitosan has been demonstrated to exhibit superior printability, degradation, and swelling properties when subjected to elevated levels of viscosity; however, it should be noted that the material has the capacity to generate elevated levels of shear stress, a factor which has been shown to compromise cell biocompatibility [133]. The utilization of nanoHAp‐chitosan‐gelatin may present certain challenges during the extrusion process, owing to the material's high mineral content. This can result in variations in homogeneity and the presence of new defects in the tooth structure [134]. As indicated in this paper, the utilization of chitosan is advantageous due to its multifaceted properties. However, it is important to note that the practical implementation of this material is not without its challenges.

### 3.4 Other Hydrogels

A naturally occurring glycosaminoglycan found in the ECM, hyaluronic acid (HA) is a promising option for periodontal tissue engineering because it is essential for cell migration, proliferation, and tissue hydration [135]. HA‐based hydrogels have been extensively studied for use as scaffolds or drug delivery systems in regenerative applications because of their high biocompatibility, biodegradability, and viscoelasticity [136]. The effectiveness of HA‐composite hydrogels in improving alveolar bone formation and lowering inflammatory responses in periodontitis models has been demonstrated by recent developments [137]. For example, a new HA/chitosan/PVA ternary hydrogel showed good physicochemical characteristics and markedly enhanced periodontal regeneration results in vivo [138]. Similarly, by encouraging collagen deposition and lowering inflammatory infiltration, pH‐responsive HA‐based hydrogels have demonstrated controlled degradation and effective immunomodulation, improving periodontal repair [139].

A semi‐synthetic hydrogel made from denatured collagen, GelMA is functionalized with methacrylate groups to enable photo‐crosslinking, which allows for adjustable mechanical properties and structural integrity [140]. GelMA scaffolds promote cell adhesion, proliferation, and remodeling because they preserve matrix metalloproteinase (MMP)‐sensitive sites and cell‐adhesive motifs (such as RGD sequence), which makes them ideal for periodontal tissue engineering [141]. According to recent research, GelMA stiffness can be adjusted to increase PDLSCs' osteogenic potential through mechanotransductive signaling pathways like extracellular signal‐regulated kinase (ERK)–Yes‐associated protein (YAP) nuclear translocation, thereby promoting bone regeneration in periodontal defects [142]. Furthermore, bioactive functionalization, such as the addition of boric acid or herbal remedies, has improved the regenerative efficacy of GelMA‐based hydrogels by enabling them to fight oxidative stress and cellular senescence in inflammatory periodontal environments [143].

Pectin is a naturally occurring polysaccharide that is mainly extracted from apple pomace and citrus peels. It gels through ionic crosslinking with divalent cations like Ca^2+^. Pectin‐based hydrogels have drawn more interest in periodontal tissue engineering because of their biocompatibility, adjustable rheological behavior, and capacity to act as carriers for bioactive substances [144, 145]. The incorporation of bioactive glass nanoparticles into ionically crosslinked pectin microspheres has been investigated recently, and the results show improved pro‐osteogenic and antibacterial properties that support periodontal regeneration [146]. Additionally, methacrylated pectin‐based hydrogels functionalized with silk fibroin or glutathione have been used in sono‐chemodynamic therapy to control reactive oxygen species (ROS) and encourage the healing of infected bone, creating opportunities for dual‐function scaffolds with both antimicrobial and regenerative properties [143].

Red algae are the source of agarose, a linear polysaccharide made up of repeating agarobiose units that, when cooled below its gelling temperature, can form thermoreversible hydrogels. It is appropriate for encasing cells or biomolecules in periodontal tissue engineering due to its capacity to produce a highly porous, inert, and mechanically stable 3D matrix [147]. Agarose hydrogels are biologically inert on their own, but they have been effectively mixed with bioactive substances to enhance cellular response and encourage tissue repair. According to a recent study, agarose‐based composite hydrogels improved periodontal ligament cell proliferation and osteogenic differentiation while preserving structural integrity for defect filling when functionalized with gelatin and growth factors [148]. Furthermore, as the most recent hydrogel engineering literature for craniofacial applications highlights, its compatibility with 3D bioprinting technologies offers significant advantages in designing customized scaffolds for periodontal defect geometries [149].

Key proteins in the coagulation cascade, fibrin and its precursor fibrinogen, have been used as biomaterials in periodontal regeneration due to their innate function in wound healing [150]. Fibrin‐based hydrogels support angiogenesis, cellular infiltration, and ECM deposition by offering a bioactive matrix that resembles the natural extracellular environment. The engineering of composite fibrin hydrogels with improved mechanical stability and bioactivity has been the focus of recent innovations [150]. For instance, chitosan‐enriched fibrin hydrogels have shown enhanced mechanical characteristics and biocompatibility, promoting the growth of dental pulp cells and preserving structural stability in damp conditions typical of the oral cavity [151]. Furthermore, in a rat model of periodontitis, a plasma‐derived fibrin hydrogel intended for regulated release of Cu and Zn ions has demonstrated notable effectiveness in stimulating alveolar bone regeneration and reducing inflammatory reactions [152].

Because of its exceptional mechanical strength, adjustable degradation, and inherent biocompatibility, silk fibroin (SF), a protein derived from Bombyx mori silk cocoons, has drawn increasing attention in periodontal and craniofacial tissue engineering [153]. SF hydrogels provide structural flexibility for encasing bioactive molecules or nanoparticles while promoting cell adhesion and proliferation. Recent studies have shown improved osteoinductive potential and antibacterial activity in enzyme‐crosslinked injectable SF/gum tragacanth hydrogels embedded with mesoporous bioactive glass nanoparticles, suggesting a potent capacity for periodontal bone regeneration [154]. Additionally, nitrogen‐doped reduced graphene oxide‐enhanced SF hydrogels have demonstrated better wound healing performance because of their increased electrical conductivity and capacity to scavenge free radicals, both of which promote faster periodontal soft and hard tissue regeneration [155]. Table 1 summarizes the hydrogels presented, separated in source, crosslinking method, key properties, applications, and limitations.

**TABLE 1:** Comparative overview of natural and semi‐synthetic hydrogels used in periodontal regeneration.

Hydrogel	Source	Crosslinking	Key properties	Applications	Limitations	Refs.
Agarose	Algal polysaccharide	Thermal gelation	Thermoreversible, mechanical stable	3D bioprinting, in vitro periodontal modeling	Biologically inert	[149]
Alginate	Brown seaweed	Ionic (Ca^2+^), covalent (MA/UV)	Biocompatibility, fast gel formation, tunable	Cell encapsulation, drug release, bioprinting	Low cell adhesion, ion‐loss in PBS	[74]
Collagen	Animal ECM (Type I)	Thermal, pH, genipin	Bioactive, cell adhesion, biodegradable	Wound healing, GTR/GBR;	Low mechanical stability, shrinking, fast degradation	[73]
Chitosan	Chitin (shells/fungi)	TPP ionic, β‐GP thermo, genipin	Cationic, antimicrobial	Periodontal gel, drug delivery	pH‐dependent, variability	[101]
Fibrin	Plasma protein	Enzymatic	Hemostatic, angiogenic, supports ECM	Injectable matrices, GTR/GBR	Fast degradation, low mechanical stability	[150, 151]
GelMA	Gelatin derivative	Methacrylation	Bioactive, tunable stiffness, 3D printing	Osteoinductive scaffolds, cell‐laden matrices	Photoinitiator, UV exposure	[140]
HA	Natural (e.g., ECM)	Chemical, enzymatic	Biocompatibility, anti‐inflammatory	Injectable, drug delivery	Fast degradation, low mechanical stability	[135, 136]
Pectin	Plant polysaccharide	Ionic (Ca^2+^), chemical	Antioxidant, mucoadhesive	Injectable, drug delivery	Low mechanical stability	[146]
Silk fibroin	Insect protein (Bombyx mori)	Enzymatic, physical, self‐assembling	Biocompatible, strong mechanical properties, tunable degradation	Injectable, bone tissue regeneration	Process complexity, batch variability	[153, 154]

β‐GP: beta‐glycerophosphate. ECM: extracellular matrix. GelMA: gelatin methacrylate. GTR/GBR: guided tissue regeneration/guided bone regeneration. HA: hyaluronic acid. UV: ultraviolet.

HA, GelMA, pectin, agarose, fibrin, and SF are just a few of the many natural and semi‐synthetic hydrogels that, when combined, provide a broad range of physicochemical and biological characteristics specifically designed for periodontal regeneration. In addition to offering bioactivity and structural support, these materials also make it possible to incorporate functional molecules or cells, which improves therapeutic results. Transforming these hydrogel systems into clinically useful treatments for periodontal tissue repair will require ongoing developments in biomaterial design and functionalization techniques.

### 3.5 Hybrid and Functionalized Hydrogels

Hybrid and functionalized hydrogels are a sophisticated class of biomaterials that combine natural and synthetic polymers, often incorporating bioactive substances, nanoparticles, or living cells [66]. These systems are designed to cooperatively replicate the intricacy of the periodontal ECM, enhance mechanical integrity, and deliver biological signals to guide tissue regeneration [14].

Natural polymers like alginate, collagen, and chitosan make up interpenetrating networks (IPNs), which present a promising way to combine advantageous qualities from each component [156]. For instance, collagen and chitosan offer better bioactivity and cellular adhesion, whereas alginate offers structural stability and ease of gelation [14]. These networks are appropriate for in situ periodontal applications because of their enhanced elasticity and degradation profiles, especially when paired with injectable delivery systems or 3D printing [157].

The osteoconductivity and mechanical reinforcement of hydrogels can be further improved by composites that include inorganic nanoparticles like nano‐hydroxyapatite (nHAp), tricalcium phosphate (TCP), or bioactive glass. These materials may also act as carriers for the long‐term release of osteoinductive cues in addition to improving mineral deposition [158]. According to recent research, 3D‐printed hybrid chitosan/alginate scaffolds loaded with nHAp improved bone and cartilage regeneration in vivo [159, 160].

A key strategy for improving regenerative results is functionalization using growth factors, stem cells, or other biologics. Molecules such as bone morphogenetic protein 2, platelet‐derived growth factor, or stem cell‐derived exosomes can be delivered spatiotemporally using functionalized hydrogels as local depots [161]. For example, in preclinical models, collagen‐alginate scaffolds functionalized with HA and stem cells showed improved periodontal ligament and alveolar bone regeneration [162, 163].

Preclinical and early‐stage clinical studies have shown the synergistic effects of combining biopolymers with nanoparticles and biologics [89]. These multipurpose systems proved capable of promoting angiogenesis, regulating inflammation, and enabling the coordinated regeneration of both soft and hard periodontal tissues [13]. Notably, alginate, collagen, and chitosan‐based injectable and self‐healing hybrid hydrogels hold promise for minimally invasive treatments that lessen surgical trauma while improving clinical results [164]. These results support the idea that hybrid and functionalized hydrogels can provide customized platforms for next‐generation treatments by bridging the translational gap between biomaterial science and clinical periodontology.

## 4 Research Gaps and Limitations

Although remarkable progress has been made in hydrogel‐based approaches for periodontal regeneration, a research gap becomes evident due to the lack of clinical studies [165]. Most current evidence is limited to in vitro tests and preclinical animal models, which, although important, cannot fully reproduce the complexity of human periodontitis [166]. For example, gelatin‐ and collagen‐based structures have shown promising results in modulating periodontal inflammation and supporting tissue growth in experimental models; however, their transition into controlled clinical settings has not yet been sufficiently explored [167, 168, 169]. Likewise, studies evaluating hydrogels incorporating growth factors or stem cell niches confirmed their ability to promote osteogenesis and cementogenesis in vivo, but none have provided solid evidence from randomized clinical trials that could demonstrate safety, long‐term stability, and clinical efficacy [170]. This lack of clinical validation restricts the possibility of immediate application of these biomaterials in daily periodontal practice.

Another important limitation involves the mechanical properties of natural polymer‐based hydrogels. While materials such as alginate, collagen, and gelatin exhibit high biocompatibility and biodegradability, they often lack adequate mechanical resistance under functional oral conditions [105]. This drawback compromises their ability to maintain structural integrity over time, especially in load‐bearing sites such as alveolar bone defects. Drug‐loaded chitosan scaffolds, for example, have demonstrated excellent biological properties but have been reported to possess low mechanical resistance and uncontrollable pore size, which may hinder predictable regenerative outcomes [171, 172]. Similarly, enzymatically crosslinked chitosan hydrogels degraded rapidly in vivo, which, although beneficial for cellular integration, raised concerns about their ability to provide sustained structural support during the critical phases of tissue regeneration [173]. The challenge of combining biodegradability with sufficient mechanical stability remains a central obstacle for the clinical translation of these biomaterials [174, 175].

A further limitation is the difficulty in reproducing the complex architecture of the periodontium using current hydrogel systems. Periodontal tissues consist of multiple compartments, including cementum, periodontal ligament, alveolar bone, and gingiva, as previously shown, each with distinct structural, mechanical, and biological requirements [176]. Although strategies employing decellularized tooth and periodontal ligament matrices within collagen hydrogels have shown the ability to stimulate the formation of new cementum and fibrous tissues, the complete integration of all periodontal components has not yet been achieved [177, 178, 179]. Likewise, advanced multifunctional hydrogels designed for sequential release of therapeutic molecules have shown improved outcomes in diabetic periodontitis models, but they still fall short of fully mimicking the hierarchical and anisotropic architecture of the native periodontium [180]. The inability to fully replicate this complexity remains a significant bottleneck, emphasizing the need for more sophisticated design strategies that integrate architectural, biomechanical, and biochemical cues [180].

Future research should therefore focus on the development of multifunctional hydrogel systems capable of simultaneously modulating inflammation, controlling infection, and promoting coordinated regeneration of periodontal tissues. Approaches incorporating controlled delivery of stem cells, bioactive molecules, extracellular vesicles, or immunomodulatory agents may represent promising strategies to enhance regenerative outcomes. Furthermore, emerging technologies such as biofabrication, in general, and more specifically, 3D bioprinting, offer new opportunities to design biomaterials with spatially controlled architecture and improved biological performance. Addressing these challenges will be essential for translating natural polymer‐based hydrogels from experimental platforms to clinically viable therapies for periodontal regeneration [181]. Overcoming these hurdles will require not only advances in biomaterial design but also in translational studies that rigorously evaluate their performance in human patients. Addressing these issues is essential for transforming current preclinical achievements into clinically reliable therapies that effectively restore periodontal health [181, 182, 183].

## 5 Design and Construction Strategies for Natural Polymer‐Based Hydrogels

The rational design of natural polymer‐based hydrogels has emerged as a key strategy for developing advanced biomaterials capable of supporting periodontal tissue regeneration [45]. Unlike conventional scaffolds, hydrogel systems can be engineered to simultaneously provide structural support, biochemical signaling, and localized delivery of therapeutic agents [184]. As a result, current research increasingly focuses on the construction strategies that govern hydrogel architecture, polymer interactions, and functionalization, enabling the development of biomimetic matrices tailored to the complex microenvironment of periodontal tissues [45, 184].

One of the fundamental aspects of hydrogel construction involves the selection and combination of suitable natural polymers [185]. Polysaccharides such as alginate and chitosan, as well as protein‐based polymers including collagen and gelatin, are widely employed due to their intrinsic biocompatibility, biodegradability, and ability to mimic key structural components of the extracellular matrix (ECM) [186]. These materials can form hydrated three‐dimensional polymer networks that support cell adhesion, proliferation, and differentiation, while also facilitating nutrient diffusion and metabolic exchange within regenerating tissues. In periodontal applications, such matrices are particularly advantageous because they can replicate aspects of the native periodontal ECM while maintaining sufficient flexibility to adapt to irregular bone defects [185, 186].

Beyond polymer selection, the structural engineering of hydrogel networks represents another crucial design parameter [184]. Modern construction strategies frequently employ composite or hybrid hydrogels in which natural polymers are combined with other polymers, bioactive ceramics, or nanoparticles to improve mechanical stability and biological functionality [187]. Interpenetrating polymer networks (IPNs), multilayer hydrogels, and composite scaffolds incorporating hydroxyapatite or bioactive glass have been widely explored to enhance osteogenic potential and mechanical resistance [185]. Such hybrid systems allow to overcome the intrinsic limitations of many natural polymers, particularly their relatively low mechanical strength and rapid degradation under physiological conditions [187].

Another important construction strategy involves the functionalization of hydrogels with bioactive agents capable of modulating the regenerative microenvironment [187]. Hydrogels can serve as reservoirs for the controlled release of antibiotics, anti‐inflammatory drugs, growth factors, or signaling molecules that regulate cellular behavior during tissue repair [186]. In the context of periodontal regeneration, this capability is particularly relevant because the inflammatory and microbial conditions associated with periodontitis often impair tissue healing [28]. By incorporating therapeutic molecules, nanoparticles, or extracellular vesicles into the hydrogel network, multifunctional systems can be created that simultaneously control infection, regulate inflammation, and stimulate osteogenic or ligament‐forming processes [187].

Finally, advances in fabrication technologies have expanded the range of construction strategies available for natural polymer hydrogels [45]. Injectable systems capable of in situ gelation are particularly attractive for periodontal therapy, as they allow minimally invasive delivery and conformal filling of irregular periodontal defects [30]. Additionally, emerging techniques such as biofabrication in general and 3D bioprinting in particular enable the creation of scaffolds with precisely controlled architectures, improving the spatial organization of cells and biomolecules within the hydrogel matrix [186]. These approaches offer promising opportunities for designing next‐generation regenerative biomaterials capable of orchestrating the coordinated regeneration of alveolar bone, periodontal ligament, and cementum.

## 6 Recent Progress in Hydrogel Crosslinking Methods

Recent advances in hydrogel design for periodontal regeneration have been strongly driven by progress in crosslinking strategies, because the junction chemistry that stabilizes the polymeric network dictates injectability, gelation kinetics, mechanical performance under oral function, degradation, and the transport of bioactive agents [66]. Hydrogels are often categorized as chemically or physically crosslinked based on the type of junctions that keep the network stable. Physically crosslinked systems rely on dynamic, non‐covalent connections and are therefore often reversible, more susceptible to environmental stimuli such as pH and temperature, and frequently physically weaker than covalently crosslinked networks [188]. In the periodontal niche, where moisture, enzymatic activity, and cyclic loading can hasten softening and disintegration if the network is not appropriately maintained, this differentiation is very crucial [189].

Current research has increased the usage of orthogonal and multi‐interaction networks in physical crosslinking, which integrate many non‐covalent patterns to increase robustness while preserving cytocompatibility [190]. In order to adjust stiffness, self‐healing, and responsiveness, ionic bonding is still a fundamental strategy for polysaccharides, supplemented by metal‐ion coordination, hydrophobic interactions, and hydrogen bonding [190]. In natural‐polymer systems, these dynamic junctions are increasingly engineered to provide “on‐demand” transitions (e.g., sol‐to‐gel behavior for minimally invasive placement), while still permitting local remodeling and diffusion‐driven release [191]. However, because physical hydrogels are frequently less robust than chemically crosslinked ones, recent designs often pair reversible interactions with a secondary stabilization step to resist oral mechanical stress [192].

Chemical crosslinking advancements have had an equally significant influence, particularly through reactions that take place in moderate, aqueous environments that are compatible with proteins and cells. In order to achieve controlled network formation and degradation, modern periodontal hydrogel systems frequently use click‐type coupling, Schiff‐base reactions, free‐radical polymerization, and phenolic oxidation chemistries [193]. Covalent crosslinking typically yields higher stability and improved mechanical properties. Notably, dynamic covalent variants (like imine‐type linkages) are being used to balance adaptability and stability, allowing for partial self‐repair and stress relaxation—features important for maintaining contact with irregular defect geometries and integrating with the periodontal ligament microenvironment [194].

The growth of photo‐crosslinking platforms, which provide temporal and spatial control over gelation and mechanics, is a significant contemporary development [195]. GelMA, a semi‐synthetic hydrogel made from denatured collagen and functionalized with methacrylate groups to facilitate photo‐crosslinking and support modifiable mechanical characteristics and structural integrity, serves as an example of this. In order to match gelation and performance to the intended delivery route and defect type, natural polymers like chitosan are increasingly being formulated to be printable and clinically manageable by utilizing multiple structuring routes, such as ionic complexation, covalent crosslinking (such as genipin and carbodiimide‐based coupling), and photo‐crosslinking [196].

Overall, the field is moving toward hybrid and multi‐step crosslinking (combining fast in situ setting with a secondary reinforcement mechanism) to better balance injectability and cytocompatibility with the mechanical demands and biochemical complexity of periodontal regeneration.

## 7 Conclusions

The development and use of hydrogels for periodontal regeneration have advanced significantly in recent years, especially those derived from natural polymers like alginate, collagen, and chitosan. According to available data, these materials can mimic important features of the natural extracellular matrix, promote cell adhesion and proliferation, and offer a flexible platform for the targeted delivery of bioactive substances. When these hydrogels have been combined with growth factors, stem cells, or other regenerative cues, preclinical studies consistently showed improved formation of cementum, periodontal ligament, and alveolar bone, highlighting their potential as key elements of next‐generation periodontal therapies.

Despite these developments, significant obstacles still need to be overcome before natural hydrogels can be completely incorporated into standard clinical procedures. Their widespread use is still limited by issues of low mechanical strength, batch‐to‐batch variability, poor handling characteristics, and rapid degradation kinetics in the complex oral environment. Furthermore, there is a scarcity of standardized protocols for rheological, mechanical, and biological characterization that would enable reliable comparisons between various systems. Moreover, there are still a few well‐designed, controlled clinical trials. In order to move from encouraging preclinical results to steady, long‐term clinical success, these problems must be resolved.

Looking ahead, smart and stimuli‐responsive hydrogels stand out as a particularly attractive avenue for periodontal regeneration. Instead of behaving as passive fillers, these systems are designed to “sense” their surroundings and react to changes in pH, enzyme levels, inflammatory mediators, or mechanical forces. In practice, this means they can release drugs when and where they are most needed and adjust their properties as healing progresses, bringing the behavior of the material much closer to the dynamics of living tissue. At the same time, 3D bioprinting is creating new opportunities to build patient‐specific constructs with controlled architecture and composition, helping to more faithfully reproduce the complex, layered organization of the periodontium. The emerging concept of 4D bioprinting, in which printed hydrogels can change shape or properties over time in a programmed way, further broadens the possibilities for creating adaptable scaffolds that evolve together with the healing tissue.

Taken together, these advances show how natural polymer‐based hydrogels have moved far beyond the role of simple carriers to become sophisticated, multifunctional platforms with real potential to change the way periodontal disease is treated. The path to clinical impact, however, will require more than innovative materials alone. Progress will depend on well‐planned translational research that links materials science, biofabrication, and stimuli‐responsive technologies with careful biological testing and robust clinical trial design. Equally important will be closer collaboration between basic scientists, periodontists, bioengineers, and regulatory bodies, so that promising concepts in the laboratory can be realistically adapted to the constraints and needs of clinical practice. If these efforts are aligned, hydrogel‐based strategies are likely to play an increasingly central role in the future, minimally invasive and more personalized management of periodontitis.

## Conflicts of Interest

The authors declare no conflicts of interest.

## Data Availability

Data sharing not applicable to this article as no datasets were generated or analyzed during the current study.

## References

[1] M. C. Bottino , V. Thomas , G. Schmidt , et al., “Recent Advances in the Development of GTR/GBR Membranes for Periodontal Regeneration—A Materials Perspective,” Dental Materials 28, no. 7 (2012): 703–721, 10.1016/j.dental.2012.04.022. 22592164

[2] G. A. N. Atia , H. K. Shalaby , M. Zehravi , et al., “Drug‐Loaded Chitosan Scaffolds for Periodontal Tissue Regeneration,” Polymers 14, no. 15 (2022): 3192, 10.3390/polym14153192. 35956708 PMC9371089

[3] X.‐Z. Yan , J. J. J. P. Van Den Beucken , X. Cai , N. Yu , J. A. Jansen , and F. Yang , “Periodontal Tissue Regeneration Using Enzymatically Solidified Chitosan Hydrogels With or Without Cell Loading,” Tissue Engineering Part A 21, no. 5–6 (2015): 1066–1076, 10.1089/ten.tea.2014.0319. 25345525 PMC4356215

[4] Q. Zhang , C. Gou , and Z. Zhang , “Biomimetic Materials: A Promising Strategy for Periodontal Tissue Engineering and Regeneration,” Frontiers in Bioengineering and Biotechnology 13 (2025): 1639170, 10.3389/fbioe.2025.1639170. 41376696 PMC12687456

[5] C. L. Salgado , A. Cochis , E. M. Varoni , and R. A. Mendes , “Editorial: The Expanding Frontiers of Stem Cells Therapy in Oral Maxillo‐Facial Engineering and Regenerative Medicine,” Frontiers in Bioengineering and Biotechnology 13 (2025): 1548950, 10.3389/fbioe.2025.1548950. 39912112 PMC11794199

[6] A. A. Ivanov , O. P. Popova , A. V. Kuznetsova , et al., “Induction of Periodontal Endogenous Regeneration by 3D Bioscaffolds,” Bulletin of Experimental Biology and Medicine 179, no. 1 (2025): 103–107, 10.1007/s10517-025-06442-7. 40682654

[7] Q. Li , D. Wang , C. Xiao , H. Wang , and S. Dong , “Advances in Hydrogels for Periodontitis Treatment,” ACS Biomaterials Science & Engineering 10, no. 5 (2024): 2742–2761, 10.1021/acsbiomaterials.4c00220. 38639082

[8] Z. Xu , J. Wang , L. Gao , and W. Zhang , “Hydrogels in Alveolar Bone Regeneration,” ACS Biomaterials Science & Engineering 10, no. 12 (2024): 7337–7351, 10.1021/acsbiomaterials.4c01359. 39571179

[9] M. Usui , S. Onizuka , T. Sato , S. Kokabu , W. Ariyoshi , and K. Nakashima , “Mechanism of Alveolar Bone Destruction in Periodontitis — Periodontal Bacteria and Inflammation,” Japanese Dental Science Review 57 (2021): 201–208, 10.1016/j.jdsr.2021.09.005. 34703508 PMC8524191

[10] Y. Gu , Y. Hu , S. Huang , et al., “CpG ODN/Mangiferin Dual Delivery Through Calcium Alginate Hydrogels Inhibits Immune‐Mediated Osteoclastogenesis and Promotes Alveolar Bone Regeneration in Mice,” Biology 12, no. 7 (2023): 976, 10.3390/biology12070976. 37508406 PMC10376397

[11] S. J. Bidarra , C. C. Barrias , and P. L. Granja , “Injectable Alginate Hydrogels for Cell Delivery in Tissue Engineering,” Acta Biomaterialia 10, no. 4 (2014): 1646–1662, 10.1016/j.actbio.2013.12.006. 24334143

[12] X. Chen , T. Wu , Y. Bu , H. Yan , and Q. Lin , “Fabrication and Biomedical Application of Alginate Composite Hydrogels in Bone Tissue Engineering: A Review,” International Journal of Molecular Sciences 25, no. 14 (2024): 7810, 10.3390/ijms25147810. 39063052 PMC11277200

[13] M. El‐Nablaway , F. Rashed , E. S. Taher , et al., “Bioactive Injectable Mucoadhesive Thermosensitive Natural Polymeric Hydrogels for Oral Bone and Periodontal Regeneration,” Frontiers in Bioengineering and Biotechnology 12 (2024): 1384326, 10.3389/fbioe.2024.1384326. 38863491 PMC11166210

[14] M. Li , J. Lv , Y. Yang , et al., “Advances of Hydrogel Therapy in Periodontal Regeneration—A Materials Perspective Review,” Gels 8, no. 10 (2022): 624, 10.3390/gels8100624. 36286125 PMC9602018

[15] Z. Zhang , F. Bi , and W. Guo , “Research Advances on Hydrogel‐Based Materials for Tissue Regeneration and Remineralization in Tooth,” Gels 9, no. 3 (2023): 245, 10.3390/gels9030245. 36975694 PMC10048036

[16] X. Lan , Y. Wang , and M. Yin , “Enhancing Periodontal Ligament Regeneration via PDLSC Delivery Using Electrospun PCL/Collagen/Cellulose Acetate Scaffolds and Collagen Hydrogel Incorporated With Curcumin‐Loaded ZIF‐8 Nanoparticles,” International Journal of Nanomedicine 20 (2025): 887–906, 10.2147/IJN.S492274. 39867310 PMC11761539

[17] J. Zhou , H. Li , S. Li , et al., “Convertible Hydrogel Injection Sequentially Regulates Diabetic Periodontitis,” ACS Biomaterials Science & Engineering 11, no. 2 (2025): 916–929, 10.1021/acsbiomaterials.4c01784. 39792458

[18] C. Liang , L. Liao , and W. Tian , “Advances Focusing on the Application of Decellularized Extracellular Matrix in Periodontal Regeneration,” Biomolecules 13, no. 4 (2023): 673, 10.3390/biom13040673. 37189420 PMC10136219

[19] A. A. Ivanov , A. V. Kuznetsova , O. P. Popova , T. I. Danilova , A. V. Latyshev , and O. O. Yanushevich , “Influence of Extracellular Matrix Components on the Differentiation of Periodontal Ligament Stem Cells in Collagen I Hydrogel,” Cells 12, no. 19 (2023): 2335, 10.3390/cells12192335. 37830549 PMC10571948

[20] M. M. Islam , M. Kumar , M. A. Mujtaba , et al., “Formulation Development, Box‐Behnken Design‐Based Optimization and Evaluation of Cisplatin‐Loaded Chitosan Nanoparticles Embedded in Mucoadhesive Buccal Film for Targeted Oral Cancer Therapy,” Journal of Pharmaceutical Innovation 20, no. 6 (2025): 276, 10.1007/s12247-025-10163-9.

[21] I. D. Zlotnikov , N. G. Belogurova , I. V. Poddubnaya , and E. V. Kudryashova , “Mucosal Adhesive Chitosan Nanogel Formulations of Antibiotics and Adjuvants (Terpenoids, Flavonoids, etc.) and Their Potential for the Treatment of Infectious Diseases of the Gastrointestinal Tract,” Pharmaceutics 15, no. 9 (2023): 2353, 10.3390/pharmaceutics15092353. 37765322 PMC10535539

[22] M. Amato , S. Santonocito , A. Polizzi , et al., “Local Delivery and Controlled Release Drugs Systems: A New Approach for the Clinical Treatment of Periodontitis Therapy,” Pharmaceutics 15, no. 4 (2023): 1312, 10.3390/pharmaceutics15041312. 37111796 PMC10143241

[23] J. Carrasco‐Sandoval , M. Aranda , K. Henríquez‐Aedo , M. Fernández , A. López‐Rubio , and M. J. Fabra , “Impact of Molecular Weight and Deacetylation Degree of Chitosan on the Bioaccessibility of Quercetin Encapsulated in Alginate/Chitosan‐Coated Zein Nanoparticles,” International Journal of Biological Macromolecules 242 (2023): 124876, 10.1016/j.ijbiomac.2023.124876. 37182618

[24] Z. Ai , D. Li , Y. Tian , Y. Wang , Y. Hou , and C. Zhang , “Dual‐Functional Natural Drug‐Loaded Responsive Hydrogel for Activating Autophagy and Regulating Immune Microenvironment in the Treatment of Diabetic Periodontitis,” Chemical Engineering Journal 521 (2025): 166999, 10.1016/j.cej.2025.166999.

[25] H. Wu , Y. Li , L. Shi , Y. Liu , and J. Shen , “New Advances in Periodontal Functional Materials Based on Antibacterial, Anti‐Inflammatory, and Tissue Regeneration Strategies,” Advanced Healthcare Materials 14, no. 9 (2025): 2403206, 10.1002/adhm.202403206. 39895157

[26] R. Khademi and M. Kharaziha , “Recent Advances in Hydrogel‐Based Platforms for Periodontal Tissue Regeneration,” Current Opinion in Biomedical Engineering 35 (2025): 100615, 10.1016/j.cobme.2025.100615.

[27] M. Chelu , M. Popa , and J. M. Calderón Moreno , “Next‐Generation Natural Hydrogels in Oral Tissue Engineering,” Pharmaceutics 17, no. 10 (2025): 1256, 10.3390/pharmaceutics17101256. 41155894 PMC12567024

[28] W. Guo , H. Dong , and X. Wang , “Emerging Roles of Hydrogel in Promoting Periodontal Tissue Regeneration and Repairing Bone Defect,” Frontiers in Bioengineering and Biotechnology 12 (2024): 1380528, 10.3389/fbioe.2024.1380528. 38720879 PMC11076768

[29] Z. Gouveia , M. Diba , B. T. Yilmaz , J. Cha , D. T. Wu , and D. J. Mooney , “Hydrogels in Periodontal and Craniofacial Regeneration: Current Applications and Next‐Generation Biomaterials,” Journal of Periodontal Research (2026): jre70059, 10.1111/jre.70059. 41563343

[30] M. A. Amiri , D. Amiri , and S. Hamedani , “Thermosensitive Hydrogels for Periodontal Regeneration: A Systematic Review of the Evidence,” Clinical and Experimental Dental Research 10, no. 6 (2024): 70029, 10.1002/cre2.70029. PMC11561135 39539029

[31] G. Yürük , Y. D. Demir , Ş. Vural , and N. S. Kehr , “Polymeric Biomaterials for Periodontal Tissue Engineering and Periodontitis,” RSC Applied Polymers 2, no. 4 (2024): 534–556, 10.1039/D4LP00001C.

[32] Q. Pan , Z. Zong , H. Li , et al., “Hydrogel Design and Applications for Periodontitis Therapy: A Review,” International Journal of Biological Macromolecules 284 (2025): 137893, 10.1016/j.ijbiomac.2024.137893. 39571840

[33] N. S. Gasner and R. S. Schure , “Periodontal Disease,” in StatPearls (StatPearls Publishing, 2025). 32119477

[34] M. X. Chen , Y. J. Zhong , Q. Q. Dong , H. M. Wong , and Y. F. Wen , “Global, Regional, and National Burden of Severe Periodontitis, 1990–2019: An Analysis of the Global Burden of Disease Study 2019,” Journal of Clinical Periodontology 48, no. 9 (2021): 1165–1188, 10.1111/jcpe.13506. 34101223

[35] D. Batista‐Cárdenas , A. Araya‐Castillo , M. P. Arias‐Campos , et al., “Association of the Severity and Progression Rate of Periodontitis With Systemic Medication Intake,” Frontiers in Oral Health 5 (2024): 1447019, 10.3389/froh.2024.1447019. 39157205 PMC11328918

[36] E. Könönen , M. Gursoy , and U. Gursoy , “Periodontitis: A Multifaceted Disease of Tooth‐Supporting Tissues,” Journal of Clinical Medicine 8, no. 8 (2019): 1135, 10.3390/jcm8081135. 31370168 PMC6723779

[37] P. Carvajal , F. C. D. A. Carrer , M. L. Galante , R. Vernal , and C. B. Solis , “Prevalence of Periodontal Diseases: Latin America and the Caribbean Consensus 2024,” Brazilian Oral Research 38, no. 1 (2024): 116, 10.1590/1807-3107bor-2024.vol38.0116. PMC11665978 39607147

[38] N. T. Hashim , R. Babiker , V. Padmanabhan , et al., “The Global Burden of Periodontal Disease: A Narrative Review on Unveiling Socioeconomic and Health Challenges,” International Journal of Environmental Research and Public Health 22, no. 4 (2025): 624, 10.3390/ijerph22040624. 40283848 PMC12027323

[39] H.‐L. Wang and L. Boyapati , ““PASS” Principles for Predictable Bone Regeneration,” Implant Dentistry 15, no. 1 (2006): 8–17, 10.1097/01.id.0000204762.39826.0f. 16569956

[40] R. Ashfaq , A. Kovács , S. Berkó , and M. Budai‐Szűcs , “Developments in Alloplastic Bone Grafts and Barrier Membrane Biomaterials for Periodontal Guided Tissue and Bone Regeneration Therapy,” International Journal of Molecular Sciences 25, no. 14 (2024): 7746, 10.3390/ijms25147746. 39062989 PMC11277074

[41] Y.‐D. Cho , K.‐H. Kim , Y.‐M. Lee , Y. Ku , and Y.‐J. Seol , “Periodontal Wound Healing and Tissue Regeneration: A Narrative Review,” Pharmaceuticals 14, no. 5 (2021): 456, 10.3390/ph14050456. 34065862 PMC8151433

[42] M. Farooq , A. W. Khan , M. S. Kim , and S. Choi , “The Role of Fibroblast Growth Factor (FGF) Signaling in Tissue Repair and Regeneration,” Cells 10, no. 11 (2021): 3242, 10.3390/cells10113242. 34831463 PMC8622657

[43] R. De Lima Barbosa , E. Stellet Lourenço , J. V. De Azevedo Dos Santos , N. Rodrigues Santiago Rocha , C. F. Mourão , and G. G. Alves , “The Effects of Platelet‐Rich Fibrin in the Behavior of Mineralizing Cells Related to Bone Tissue Regeneration—A Scoping Review of In Vitro Evidence,” Journal of Functional Biomaterials 14, no. 10 (2023): 503, 10.3390/jfb14100503. 37888168 PMC10607127

[44] S. Norouzi , N. Saveh Shemshaki , E. Norouzi , et al., “Recent Advances in Biomaterials for Tissue‐Engineered Constructs: Essential Factors and Engineering Techniques,” Materials Today Chemistry 37 (2024): 102016, 10.1016/j.mtchem.2024.102016.

[45] H. Sun , J. Luan , and S. Dong , “Hydrogels Promote Periodontal Regeneration,” Frontiers in Bioengineering and Biotechnology 12 (2024): 1411494, 10.3389/fbioe.2024.1411494. 38827033 PMC11140061

[46] Y. Pei , Y. Wang , J. Chen , et al., “Bionic Nanostructures Create Mechanical Signals to Mediate the Composite Structural Bone Regeneration Through Multi‐System Regulation,” Advanced Science 12, no. 31 (2025): 02299, 10.1002/advs.202502299. PMC12376681 40464259

[47] X. Yang , M. Nagao , and C.‐H. Zhou , “Editorial: Biomaterials and Biological Regulation for Bone Tissue Remodeling and Regeneration,” Frontiers in Bioengineering and Biotechnology 13 (2025): 1582215, 10.3389/fbioe.2025.1582215. 40242356 PMC11999967

[48] B. Uysal , U. S. K. Madduma‐Bandarage , H. G. Jayasinghe , and S. Madihally , “3D‐Printed Hydrogels From Natural Polymers for Biomedical Applications: Conventional Fabrication Methods, Current Developments, Advantages, and Challenges,” Gels 11, no. 3 (2025): 192, 10.3390/gels11030192. 40136897 PMC11942323

[49] R. D. Kasai , D. Radhika , S. Archana , et al., “A Review on Hydrogels Classification and Recent Developments in Biomedical Applications,” International Journal of Polymeric Materials and Polymeric Biomaterials 72, no. 13 (2023): 1059–1069, 10.1080/00914037.2022.2075872.

[50] S. Sen , R. Sahu , T. K. Dua , P. Paul , and G. Nandi , “Advancements of Multifunctional Hydrogels in Treating Periodontal Diseases: A Concise Review,” Mater 8 (2025): 100825, 10.1016/j.nxmate.2025.100825.

[51] M. Neumann , G. Di Marco , D. Iudin , et al., “Stimuli‐Responsive Hydrogels: The Dynamic Smart Biomaterials of Tomorrow,” Macromolecules 56, no. 21 (2023): 8377–8392, 10.1021/acs.macromol.3c00967. 38024154 PMC10653276

[52] P. Sánchez‐Cid , M. Jiménez‐Rosado , A. Romero , and V. Pérez‐Puyana , “Novel Trends in Hydrogel Development for Biomedical Applications: A Review,” Polymers 14, no. 15 (2022): 3023, 10.3390/polym14153023. 35893984 PMC9370620

[53] M. Bustamante‐Torres , D. Romero‐Fierro , B. Arcentales‐Vera , K. Palomino , H. Magaña , and E. Bucio , “Hydrogels Classification According to the Physical or Chemical Interactions and as Stimuli‐Sensitive Materials,” Gels 7, no. 4 (2021): 182, 10.3390/gels7040182. 34842654 PMC8628675

[54] X. Ding , L. Fan , L. Wang , M. Zhou , Y. Wang , and Y. Zhao , “Designing Self‐Healing Hydrogels for Biomedical Applications,” Materials Horizons 10, no. 10 (2023): 3929–3947, 10.1039/D3MH00891F. 37577809

[55] W. Wang , Y. Zhang , and W. Liu , “Bioinspired Fabrication of High Strength Hydrogels From Non‐Covalent Interactions,” Progress in Polymer Science 71 (2017): 1–25, 10.1016/j.progpolymsci.2017.04.001.

[56] M. M. Ribeiro , M. Simões , C. Vitorino , and F. Mascarenhas‐Melo , “Physical Crosslinking of Hydrogels: The Potential of Dynamic and Reversible Bonds in Burn Care,” Coordination Chemistry Reviews 542 (2025): 216868, 10.1016/j.ccr.2025.216868.

[57] P. Yu , B. Chen , B. Xie , et al., “Recent Advances in Oral Hydrogel Drug Delivery System for Disease Treatment,” Chinese Chemical Letters (2025): 111695, 10.1016/j.cclet.2025.111695.

[58] V. G. Muir and J. A. Burdick , “Chemically Modified Biopolymers for the Formation of Biomedical Hydrogels,” Chemical Reviews 121, no. 18 (2021): 10908–10949, 10.1021/acs.chemrev.0c00923. 33356174 PMC8943712

[59] S. Ran , L. Xue , X. Wei , et al., “Recent Advances in Injectable Hydrogel Therapies for Periodontitis,” Journal of Materials Chemistry B 12, no. 25 (2024): 6005–6032, 10.1039/D3TB03070A. 38869470

[60] C. Shen , Y. Han , H. Xiong , et al., “Multifunctional Hydrogel Scaffolds Based on Polysaccharides and Polymer Matrices Promote Bone Repair: A Review,” International Journal of Biological Macromolecules 294 (2025): 139418, 10.1016/j.ijbiomac.2024.139418. 39765302

[61] X. Li and Y. Xiong , “Application of “Click” Chemistry in Biomedical Hydrogels,” ACS Omega 7, no. 42 (2022): 36918–36928, 10.1021/acsomega.2c03931. 36312409 PMC9608400

[62] M. Wu , L. Han , B. Yan , and H. Zeng , “Self‐Healing Hydrogels Based on Reversible Noncovalent and Dynamic Covalent Interactions: A Short Review,” Supramolecular Materials 2 (2023): 100045, 10.1016/j.supmat.2023.100045.

[63] N. Ranganathan , R. Joseph Bensingh , M. Abdul Kader , and S. K. Nayak , “Synthesis and Properties of Hydrogels Prepared by Various Polymerization Reaction Systems,” in Cellulose‐Based SuperabsorbentHydrogels, ed. I. H. Mondal (Springer International Publishing, 2018), 1–25, 10.1007/978-3-319-76573-0_18-1.

[64] J. Cheng , J. Liu , M. Li , et al., “Hydrogel‐Based Biomaterials Engineered From Natural‐Derived Polysaccharides and Proteins for Hemostasis and Wound Healing,” Frontiers in Bioengineering and Biotechnology 9 (2021): 780187, 10.3389/fbioe.2021.780187. 34881238 PMC8645981

[65] N. G. Fischer , I. J. De Souza Araújo , A. Daghrery , et al., “Guidance on Biomaterials for Periodontal Tissue Regeneration: Fabrication Methods, Materials and Biological Considerations,” Dental Materials 41, no. 3 (2025): 283–305, 10.1016/j.dental.2024.12.019. 39794220 PMC13022934

[66] B. Yin , J. M. Dodda , S. H. D. Wong , et al., “Smart Injectable Hydrogels for Periodontal Regeneration: Recent Advancements in Biomaterials and Biofabrication Strategies,” Materials Today Bio 32 (2025): 101855, 10.1016/j.mtbio.2025.101855. PMC12145717 40487163

[67] W. Wang , A. Wang , G. Hu , et al., “Potential of an Aligned Porous Hydrogel Scaffold Combined With Periodontal Ligament Stem Cells or Gingival Mesenchymal Stem Cells to Promote Tissue Regeneration in Rat Periodontal Defects,” ACS Biomaterials Science & Engineering 9, no. 4 (2023): 1961–1975, 10.1021/acsbiomaterials.2c01440. 36942823

[68] M. Mobaraki , M. Ghaffari , A. Yazdanpanah , Y. Luo , and D. K. Mills , “Bioinks and Bioprinting: A Focused Review,” Bioprinting 18 (2020): e00080, 10.1016/j.bprint.2020.e00080.

[69] N. D. Almeida , C. A. Carneiro , A. C. De Marco , V. C. Porto , and R. França , “3D Bioprinting Techniques and Bioinks for Periodontal Tissues Regeneration—A Literature Review,” Biomimetics 9, no. 8 (2024): 480, 10.3390/biomimetics9080480. 39194459 PMC11352156

[70] H. Chen , Y. Wang , Y. Lai , et al., “Advances of 3D Bioprinting Technology for Periodontal Tissue Regeneration,” Iscience 28, no. 6 (2025): 112532, 10.1016/j.isci.2025.112532. 40487440 PMC12141959

[71] Y. Wang , W. Geng , Y. Yang , et al., “Engineered Self‐Assembling Hydrogel Systems for Advanced Guided Bone Regeneration: Structural Optimization and Biofunctional Modulation,” Journal of Nanobiotechnology 23, no. 1 (2025): 720, 10.1186/s12951-025-03761-9. 41254726 PMC12628861

[72] Y. Liu , C. Liu , C. Wang , et al., “Treatment of Periodontal Inflammation in Diabetic Rats With IL‐1ra Thermosensitive Hydrogel,” International Journal of Molecular Sciences 23, no. 22 (2022): 13939, 10.3390/ijms232213939. 36430410 PMC9693501

[73] I. J. De Souza Araújo , R. S. Perkins , M. M. Ibrahim , G. T.‐J. Huang , and W. Zhang , “Bioprinting PDLSC‐Laden Collagen Scaffolds for Periodontal Ligament Regeneration,” ACS Applied Materials & Interfaces 16, no. 44 (2024): 59979–59990, 10.1021/acsami.4c13830. 39467547 PMC11551894

[74] J. Tan , Y. Luo , Y. Guo , et al., “Development of Alginate‐Based Hydrogels: Crosslinking Strategies and Biomedical Applications,” International Journal of Biological Macromolecules 239 (2023): 124275, 10.1016/j.ijbiomac.2023.124275. 37011751

[75] J. Liu , S. Yang , X. Li , Q. Yan , M. J. T. Reaney , and Z. Jiang , “Alginate Oligosaccharides: Production, Biological Activities, and Potential Applications,” Comprehensive Reviews in Food Science and Food Safety 18, no. 6 (2019): 1859–1881, 10.1111/1541-4337.12494. 33336967

[76] H. Zhang , J. Cheng , and Q. Ao , “Preparation of Alginate‐Based Biomaterials and Their Applications in Biomedicine,” Marine Drugs 19, no. 5 (2021): 264, 10.3390/md19050264. 34068547 PMC8150954

[77] Y. Ren , Q. Wang , W. Xu , et al., “Alginate‐Based Hydrogels Mediated Biomedical Applications: A Review,” International Journal of Biological Macromolecules 279 (2024): 135019, 10.1016/j.ijbiomac.2024.135019. 39182869

[78] L. Chen , R. Shen , S. Komasa , et al., “Drug‐Loadable Calcium Alginate Hydrogel System for Use in Oral Bone Tissue Repair,” International Journal of Molecular Sciences 18, no. 5 (2017): 989, 10.3390/ijms18050989. 28481253 PMC5454902

[79] Y. Sun , Z. Zhao , Q. Qiao , et al., “Injectable Periodontal Ligament Stem Cell‐Metformin‐Calcium Phosphate Scaffold for Bone Regeneration and Vascularization in Rats,” Dental Materials 39, no. 10 (2023): 872–885, 10.1016/j.dental.2023.07.008. 37574338

[80] L. Wang , P. Wang , M. D. Weir , M. A. Reynolds , L. Zhao , and H. H. K. Xu , “Hydrogel Fibers Encapsulating Human Stem Cells in an Injectable Calcium Phosphate Scaffold for Bone Tissue Engineering,” Biomedical Materials 11, no. 6 (2016): 065008, 10.1088/1748-6041/11/6/065008. 27811389 PMC5382017

[81] T. Boontheekul , H.‐J. Kong , and D. J. Mooney , “Controlling Alginate Gel Degradation Utilizing Partial Oxidation and Bimodal Molecular Weight Distribution,” Biomaterials 26, no. 15 (2005): 2455–2465, 10.1016/j.biomaterials.2004.06.044. 15585248

[82] H. Chen , G. Song , T. Xu , et al., “Biomaterial Scaffolds for Periodontal Tissue Engineering,” Journal of Functional Biomaterials 15, no. 8 (2024): 233, 10.3390/jfb15080233. 39194671 PMC11355167

[83] S. Ansari , S. Pouraghaei Sevari , C. Chen , P. Sarrion , and A. Moshaverinia , “RGD‐Modified Alginate–GelMA Hydrogel Sheet Containing Gingival Mesenchymal Stem Cells: A Unique Platform for Wound Healing and Soft Tissue Regeneration,” ACS Biomaterials Science & Engineering 7, no. 8 (2021): 3774–3782, 10.1021/acsbiomaterials.0c01571. 34082525

[84] F. Bashir , A. Afzaal , Shahnaz , et al., “Eco‐Friendly Development of Intrinsically Antibacterial and Mechanically Robust Self‐Healing Hydrogels Using Alginate and Oval Proteins: Advancing Periodontitis Treatment,” European Polymer Journal 220 (2024): 113423, 10.1016/j.eurpolymj.2024.113423.

[85] X. Tan , S. Liu , X. Hu , et al., “Near‐Infrared‐Enhanced Dual Enzyme‐Mimicking Ag–TiO_2–_ * _x_ *@Alginate Microspheres With Antibactericidal and Oxygeneration Abilities to Treat Periodontitis,” ACS Applied Materials & Interfaces 15, no. 1 (2023): 391–406, 10.1021/acsami.2c17065. 36562459

[86] X. Lu , S. Hu , Z. Zhang , et al., “A pH‐Sensitive CuHP Composite Hydrogel Featuring Antibacterial, Antioxidant and Osteogenic Properties for Treating Diabetic Periodontitis,” Regenerative Biomaterials 12 (2025): rbaf065, 10.1093/rb/rbaf065. 40979829 PMC12448283

[87] A.‐E. Segneanu , L. E. Bejenaru , C. Bejenaru , et al., “Advancements in Hydrogels: A Comprehensive Review of Natural and Synthetic Innovations for Biomedical Applications,” Polymers 17, no. 15 (2025): 2026, 10.3390/polym17152026. 40808075 PMC12349326

[88] M. Zussman and M. Zilberman , “Injectable Metronidazole‐Eluting Gelatin‐Alginate Hydrogels for Local Treatment of Periodontitis,” Journal of Biomaterials Applications 37, no. 1 (2022): 166–179, 10.1177/08853282221079458. 35341363

[89] F. Xu , T. Deng , W. Li , et al., “A Sequential Sustained‐Release Hydrogel With Potent Antimicrobial, Anti‐Inflammatory, and Osteogenesis‐Promoting Properties for the Treatment of Periodontitis,” Chemical Engineering Journal 477 (2023): 147195, 10.1016/j.cej.2023.147195.

[90] G. Miao , L. Liang , W. Li , et al., “3D Bioprinting of a Bioactive Composite Scaffold for Cell Delivery in Periodontal Tissue Regeneration,” Biomolecules 13, no. 7 (2023): 1062, 10.3390/biom13071062. 37509098 PMC10377653

[91] Y.‐T. Chang , C.‐C. Lai , and D.‐J. Lin , “Collagen Scaffolds Laden With Human Periodontal Ligament Fibroblasts Promote Periodontal Regeneration in SD Rat Model,” Polymers 15, no. 12 (2023): 2649, 10.3390/polym15122649. 37376295 PMC10305005

[92] T. Zhou , S. Chen , X. Ding , Z. Hu , L. Cen , and X. Zhang , “Fabrication and Characterization of Collagen/PVA Dual‐Layer Membranes for Periodontal Bone Regeneration,” Frontiers in Bioengineering and Biotechnology 9 (2021): 630977, 10.3389/fbioe.2021.630977. 34178953 PMC8219956

[93] B. Gurumurthy , P. Pal , J. A. Griggs , and A. V. Janorkar , “Optimization of Collagen‐Elastin‐Like Polypeptide‐Bioglass Scaffold Composition for Osteogenic Differentiation of Adipose‐Derived Stem Cells,” Materialia 9 (2020): 100572, 10.1016/j.mtla.2019.100572. PMC7055731 32133439

[94] D. Yang , D. He , F. Yang , et al., “Advances in Harnessing Biological Macromolecules for Periodontal Tissue Regeneration: A Review,” International Journal of Biological Macromolecules 311 (2025): 144031, 10.1016/j.ijbiomac.2025.144031. 40345296

[95] T. Momose , H. Miyaji , A. Kato , et al., “Collagen Hydrogel Scaffold and Fibroblast Growth Factor‐2 Accelerate Periodontal Healing of Class II Furcation Defects in Dog,” Open Dentistry Journal 10, no. 1 (2016): 347–359, 10.2174/1874210601610010347. 27583044 PMC4974830

[96] S. Abraham , P. Gupta , K. Govarthanan , S. Rao , and T. S. Santra , “Direction‐Oriented Fiber Guiding With a Tunable Tri‐Layer‐3D Scaffold for Periodontal Regeneration,” RSC Advances 14, no. 28 (2024): 19806–19822, 10.1039/D4RA01459F. 38899033 PMC11186324

[97] D. A. Wijayanti , G. N. K. A. Wirajaya , N. H. Pratiwi , V. M. Karina , and K. Murdiastuti , “Combination of Collagen‐Chitosan Hydrogel and Injectable Platelet‐Rich Fibrin as a Biomaterial for Bone Regeneration: Characterization and Growth Factor Release Pattern,” European Journal of Dentistry (2025): 137–145, 10.1055/s-0045-1809144. 40403773 PMC12890416

[98] K. Sidharta , S. Suryono , M. A. Pritia , and K. Murdiastuti , “Hydrogel and Injectable Platelet‐Rich Fibrin: A Synergistic Approach to Osteogenesis,” European Journal of Dentistry (2025): 1–317, 10.1055/s-0045-1809528. PMC13160616 40550488

[99] Y. Feng and H.‐P. Li , “Optimizing Collagen‐Based Biomaterials for Periodontal Regeneration: Clinical Opportunities and Challenges,” Frontiers in Bioengineering and Biotechnology 12 (2024): 1469733, 10.3389/fbioe.2024.1469733. 39703793 PMC11655217

[100] M. H. Malik , L. Shahzadi , R. Batool , et al., “Thyroxine‐Loaded Chitosan/Carboxymethyl Cellulose/Hydroxyapatite Hydrogels Enhance Angiogenesis in in‐Ovo Experiments,” International Journal of Biological Macromolecules 145 (2020): 1162–1170, 10.1016/j.ijbiomac.2019.10.043. 31730970

[101] D. Lauritano , L. Limongelli , G. Moreo , G. Favia , and F. Carinci , “Nanomaterials for Periodontal Tissue Engineering: Chitosan‐Based Scaffolds. A Systematic Review,” Nanomaterials 10, no. 4 (2020): 605, 10.3390/nano10040605. 32218206 PMC7221778

[102] C.‐L. Huang , H.‐Y. Huang , Y.‐C. Lu , C.‐J. Cheng , and T.‐M. Lee , “Development of a Flexible Film Made of Polyvinyl Alcohol With Chitosan Based Thermosensitive Hydrogel,” Journal of Dental Sciences 18, no. 2 (2023): 822–832, 10.1016/j.jds.2023.01.007. 37021246 PMC10068578

[103] T.‐M. Lee , K.‐C. Chen , Y.‐C. Lu , C.‐J. Cheng , H.‐Y. Huang , and C.‐L. Huang , “Addition of Bioglass Particles to Flexible Polyvinyl Alcohol–Chitosan Hydrogel Films Strengthens Their Mechanical Properties and Osteoblast Cell Activity,” Emergent Materials 8, no. 6 (2025): 4651–4663, 10.1007/s42247-025-01114-8.

[104] R. F. Pereira , C. C. Barrias , P. J. Bártolo , and P. L. Granja , “Cell‐Instructive Pectin Hydrogels Crosslinked via Thiol‐Norbornene Photo‐Click Chemistry for Skin Tissue Engineering,” Acta Biomaterialia 66 (2018): 282–293, 10.1016/j.actbio.2017.11.016. 29128530

[105] T. Vieira De Souza , S. M. Malmonge , and A. R. Santos , “Development of a Chitosan and Hyaluronic Acid Hydrogel With Potential for Bioprinting Utilization: a Preliminary Study,” Journal of Biomaterials Applications 36, no. 2 (2021): 358–371, 10.1177/08853282211024164. 34102923

[106] A. M. Badr , H. K. Shalaby , M. A. Awad , and M. A. Hashem , “Assessment of Bone Morphogenetic Protein‐7 Loaded Chitosan/β‐Glycerophosphate Hydrogel on Periodontium Tissues Regeneration of Class III Furcation Defects,” Saudi Dental Journal 35, no. 6 (2023): 760–767, 10.1016/j.sdentj.2023.05.027. 37817788 PMC10562118

[107] S. Zang , R. Mu , F. Chen , et al., “Injectable Chitosan/β‐Glycerophosphate Hydrogels With Sustained Release of BMP‐7 and Ornidazole in Periodontal Wound Healing of Class III Furcation Defects,” Materials Science and Engineering: C 99 (2019): 919–928, 10.1016/j.msec.2019.02.024. 30889766

[108] M. S. Moreira , G. Sarra , G. L. Carvalho , et al., “Physical and Biological Properties of a Chitosan Hydrogel Scaffold Associated to Photobiomodulation Therapy for Dental Pulp Regeneration: An *In Vitro* and *In Vivo* Study,” BioMed Research International 2021, no. 1 (2021): 6684667, 10.1155/2021/6684667. 33575339 PMC7857869

[109] N. López‐Valverde , A. López‐Valverde , M. P. Cortés , C. Rodríguez , B. Macedo De Sousa , and J. M. Aragoneses , “Bone Quantification Around Chitosan‐Coated Titanium Dental Implants: A Preliminary Study by Micro‐CT Analysis in Jaw of a Canine Model,” Frontiers in Bioengineering and Biotechnology 10 (2022): 858786, 10.3389/fbioe.2022.858786. 35464727 PMC9023049

[110] S. Husain , K. H. Al‐Samadani , S. Najeeb , et al., “Chitosan Biomaterials for Current and Potential Dental Applications,” Materials 10, no. 6 (2017): 602, 10.3390/ma10060602. 28772963 PMC5553419

[111] Q. Wang , S. Tang , F. Guo , et al., “Injectable CuS‐Loaded Carboxymethyl and Sulfonated Chitosan Hydrogel With Antibacterial and Self‐Healing Properties Promoting Periodontal Tissue Regeneration,” International Journal of Biological Macromolecules 310 (2025): 143205, 10.1016/j.ijbiomac.2025.143205. 40246111

[112] Y. Karimi , M. Rashidipour , M. Iranzadasl , M. H. Ahmadi , M. M. Sarabi , and F. Farzaneh , “Biofilm Targeting With Chitosan‐Based Nanohydrogel Containing Quercus Infectoria G. Olivier Extract against Streptococcus Mutans: New Formulations of a Traditional Natural Product,” BMC Complementary Medicine and Therapies 24, no. 1 (2024): 398, 10.1186/s12906-024-04696-8. 39543581 PMC11566397

[113] M. Pourhajibagher , A. R. Rokn , M. Rostami‐Rad , H. R. Barikani , and A. Bahador , “Monitoring of Virulence Factors and Metabolic Activity in Aggregatibacter Actinomycetemcomitans Cells Surviving Antimicrobial Photodynamic Therapy via Nano‐Chitosan Encapsulated Indocyanine Green,” Frontiers in Physics 6 (2018): 124, 10.3389/fphy.2018.00124.

[114] A. Ressler , “Chitosan‐Based Biomaterials for Bone Tissue Engineering Applications: A Short Review,” Polymers 14, no. 16 (2022): 3430, 10.3390/polym14163430. 36015686 PMC9416295

[115] G. Iviglia , C. Cassinelli , E. Torre , F. Baino , M. Morra , and C. Vitale‐Brovarone , “Novel Bioceramic‐Reinforced Hydrogel for Alveolar Bone Regeneration,” Acta Biomaterialia 44 (2016): 97–109, 10.1016/j.actbio.2016.08.012. 27521494

[116] C. Zhang , Y. Fei , M. Li , et al., “Chitosan‐P407‐PNIPAM Hydrogel Loaded With AgNPs/Lipid Complex for Antibacterial, Inflammation Regulation and Alveolar Bone Regeneration in Periodontitis Treatment,” International Journal of Biological Macromolecules 307 (2025): 142080, 10.1016/j.ijbiomac.2025.142080. 40107529

[117] M. Sheridan , C. Winters , F. Zamboni , and M. N. Collins , “Biomaterials: Antimicrobial Surfaces in Biomedical Engineering and Healthcare,” Current Opinion in Biomedical Engineering 22 (2022): 100373, 10.1016/j.cobme.2022.100373.

[118] Q. Wang , X. Feng , H. Xu , G. Guo , Y. Li , and Q. Zhang , “Recent Progress of Antibacterial Hydrogel Materials for Biomedical Applications,” Journal of Materials Chemistry C 11, no. 38 (2023): 12848–12876, 10.1039/D3TC02166A.

[119] M. M. Ammar , G. H. Waly , S. H. Saniour , and T. A. Moussa , “Growth Factor Release and Enhanced Encapsulated Periodontal Stem Cells Viability by Freeze‐Dried Platelet Concentrate Loaded Thermo‐Sensitive Hydrogel for Periodontal Regeneration,” Saudi Dental Journal 30, no. 4 (2018): 355–364, 10.1016/j.sdentj.2018.06.002. 30202174 PMC6128323

[120] S. J. Dangaria , Y. Ito , C. Walker , R. Druzinsky , X. Luan , and T. G. H. Diekwisch , “Extracellular Matrix‐Mediated Differentiation of Periodontal Progenitor Cells,” Differentiation 78, no. 2–3 (2009): 79–90, 10.1016/j.diff.2009.03.005. 19433344 PMC2744845

[121] T. Nagayasu‐Tanaka , J. Anzai , S. Takaki , et al., “Action Mechanism of Fibroblast Growth Factor‐2 (FGF‐2) in the Promotion of Periodontal Regeneration in Beagle Dogs,” PLoS One 10, no. 6 (2015): 0131870, 10.1371/journal.pone.0131870. PMC4488280 26120833

[122] S. Yamano , K. Haku , T. Yamanaka , et al., “The Effect of a Bioactive Collagen Membrane Releasing PDGF or GDF‐5 on Bone Regeneration,” Biomaterials 35, no. 8 (2014): 2446–2453, 10.1016/j.biomaterials.2013.12.006. 24388383

[123] W. Dobrzyński , P. J. Piszko , J. Kiryk , et al., “Dental Resin Composites Modified With Chitosan: A Systematic Review,” Marine Drugs 23, no. 5 (2025): 199, 10.3390/md23050199. 40422789 PMC12113295

[124] C. Zúñiga‐López , K. Márquez‐Pérez , L. Argueta‐Figueroa , M. Bautista‐Hernández , and R. Torres‐Rosas , “Chitosan for the Treatment of Inflammation of the Oral Mucosa: A Systematic Review,” Oral Medicine and Pathology 29 (2024): e9–e17, 10.4317/medoral.25987. PMC10765333 37992146

[125] N. Krishna and N. Lolayekar , “Application of Chitosan Biomaterials in Dentistry—A Narrative Review,” Journal of Health and Allied Sciences 14, no. 02 (2024): 152–156, 10.1055/s-0043-1768591.

[126] R. Mascarenhas , S. Hegde , and N. Manaktala , “Chitosan Nanoparticle Applications in Dentistry: A Sustainable Biopolymer,” Frontiers in Chemistry 12 (2024): 1362482, 10.3389/fchem.2024.1362482. 38660569 PMC11039901

[127] I. Pandiyan , P. K. Rathinavelu , M. I. Arumugham , S. D , and A. Balasubramaniam , “Efficacy of Chitosan and Chlorhexidine Mouthwash on Dental Plaque and Gingival Inflammation: A Systematic Review,” Cureus 14 (2022): 23318, 10.7759/cureus.23318. PMC9014838 35464533

[128] M. Lazaridou , D. N. Bikiaris , and D. A. Lamprou , “3D Bioprinted Chitosan‐Based Hydrogel Scaffolds in Tissue Engineering and Localised Drug Delivery,” Pharmaceutics 14, no. 9 (2022): 1978, 10.3390/pharmaceutics14091978. 36145727 PMC9500618

[129] P. Maturavongsadit , L. K. Narayanan , P. Chansoria , R. Shirwaiker , and S. R. Benhabbour , “Cell‐Laden Nanocellulose/Chitosan‐Based Bioinks for 3D Bioprinting and Enhanced Osteogenic Cell Differentiation,” ACS Applied Bio Materials 4, no. 3 (2021): 2342–2353, 10.1021/acsabm.0c01108. 35014355

[130] E. Yuce‐Erarslan , R. Tutar , B. İzbudak , et al., “Photo‐Crosslinkable Chitosan and Gelatin‐Based Nanohybrid Bioinks for Extrusion‐Based 3D‐Bioprinting,” International Journal of Polymeric Materials and Polymeric Biomaterials 72, no. 1 (2023): 1–12, 10.1080/00914037.2021.1981322.

[131] H. M. Butler , E. Naseri , D. S. MacDonald , R. A. Tasker , and A. Ahmadi , “Investigation of Rheology, Printability, and Biocompatibility of N,O‐Carboxymethyl Chitosan and Agarose Bioinks for 3D Bioprinting of Neuron Cells,” Materialia 18 (2021): 101169, 10.1016/j.mtla.2021.101169.

[132] S. Suryani , A. Chaerunisaa , I. M. Joni , et al., “The Chemical Modification to Improve Solubility of Chitosan and Its Derivatives Application, Preparation Method, Toxicity as a Nanoparticles,” Nanotechnology, Science and Applications 17 (2024): 41–57, 10.2147/NSA.S450026. 38469157 PMC10926861

[133] F. Mohabatpour , X. Duan , Z. Yazdanpanah , et al., “Bioprinting of Alginate‐Carboxymethyl Chitosan Scaffolds for Enamel Tissue Engineering In Vitro,” Biofabrication 15, no. 1 (2023): 015022, 10.1088/1758-5090/acab35. 36583240

[134] N. A. Harsas , E. W. Bachtiar , L. R. Amir , et al., “Bone Graft Paste Nanohydroxyapatite Chitosan‐Gelatin (nHA/KG) for Periodontal Regeneration: Study on Three‐Dimensional Cell Culture,” European Journal of Dentistry 19, no. 04 (2025): 1035–1045, 10.1055/s-0044-1800826. 40073987 PMC12494428

[135] H. Ye , R. Zhang , C. Zhang , Y. Xia , and L. Jin , “Advances in Hyaluronic Acid: Bioactivity, Complexed Biomaterials and Biological Application: A Review,” Asian Journal of Surgery 48, no. 1 (2025): 49–61, 10.1016/j.asjsur.2024.08.100. 39217010

[136] M. Dovedytis , Z. J. Liu , and S. Bartlett , “Hyaluronic Acid and Its Biomedical Applications: A Review,” Engineered Regeneration 1 (2020): 102–113, 10.1016/j.engreg.2020.10.001.

[137] Z. Hu , X. Lv , H. Zhang , et al., “An Injectable Gel Based on Photo‐Cross‐Linkable Hyaluronic Acid and Mesoporous Bioactive Glass Nanoparticles for Periodontitis Treatment,” International Journal of Biological Macromolecules 257 (2024): 128596, 10.1016/j.ijbiomac.2023.128596. 38052282

[138] J. K. Ghonima , M. E.‐D. El‐Rashidy , G. Kotry , et al., “A Novel Composite Hyaluronic Acid/Chitosan/Polyvinyl Alcohol Ternary Hydrogel Scaffold for Periodontal Regeneration: An Experimental Study,” Preprint *BioRxiv* 8 (2025), 10.21203/rs.3.rs-7023827/v1. PMC13383553 42472801

[139] Y. Zheng , T. Zhang , and M. Guo , “Ph‐Responsive Immunomodulatory Hydrogel for Periodontitis,” International Dental Journal 75 (2025): 105088, 10.1016/j.identj.2025.105088.

[140] M. Sun , X. Sun , Z. Wang , S. Guo , G. Yu , and H. Yang , “Synthesis and Properties of Gelatin Methacryloyl (GelMA) Hydrogels and Their Recent Applications in Load‐Bearing Tissue,” Polymers 10, no. 11 (2018): 1290, 10.3390/polym10111290. 30961215 PMC6401825

[141] M. C. Bottino , D. Pankajakshan , and J. E. Nör , “Advanced Scaffolds for Dental Pulp and Periodontal Regeneration,” Dental Clinics of North America 61, no. 4 (2017): 689–711, 10.1016/j.cden.2017.06.009. 28886764 PMC5657339

[142] Y. Fu , Y. Wang , B. Cheng , R. Zou , and W. Wan , “Substrate Stiffness Regulates the Osteogenesis of PDLSCs via ERK‐Mediated YAP Nuclear Translocation,” International Dental Journal 75, no. 6 (2025): 103852, 10.1016/j.identj.2025.103852. 41075461 PMC12547731

[143] Z. Wang , Y. Huang , X. Liu , et al., “ROS‐Responsive Nanoplatform Reverses Periodontal Senescence via Dual‐Targeted Efferocytosis Remodeling and Mitochondrial Homeostasis Restoration,” Chemical Engineering Journal 525 (2025): 170411, 10.1016/j.cej.2025.170411.

[144] S. C. Neves , A. Sousa , D. S. Nascimento , et al., “A Hybrid Construct With Tailored 3D Structure for Directing Pre‐Vascularization in Engineered Tissues,” Materials Today Bio 29 (2024): 101291, 10.1016/j.mtbio.2024.101291. PMC11492604 39435373

[145] R. F. Pereira , B. N. Lourenço , P. J. Bártolo , and P. L. Granja , “Bioprinting a Multifunctional Bioink to Engineer Clickable 3D Cellular Niches With Tunable Matrix Microenvironmental Cues,” Advanced Healthcare Materials 10, no. 2 (2021): 2001176, 10.1002/adhm.202001176. 33135399

[146] S. Qiu , X. Xu , Z. Feng , et al., “Bioactive Glass‐Laden Ionically Crosslinked Pectin Microspheres With Pro‐Osteogenic and Antibacterial Activities for Periodontal Regeneration,” Ceramics International 51, no. 22 (2025): 35323–35336, 10.1016/j.ceramint.2025.05.253.

[147] F. Jiang , X.‐W. Xu , F.‐Q. Chen , et al., “Extraction, Modification and Biomedical Application of Agarose Hydrogels: A Review,” Marine Drugs 21, no. 5 (2023): 299, 10.3390/md21050299. 37233493 PMC10220669

[148] N. İ. Büyük , D. Aksu , F. N. Kök , and G. Torun Köse , “Hydrogels Used in Cartilage Treatment,” in Hydrogels and Bioinks in Tissue Engineering, N. Hasirci and V. Hasirci ed. (Springer Nature Switzerland, 2025), 259–277, 10.1007/978-3-031-90966-5_13.

[149] P. Zarrintaj , S. Manouchehri , Z. Ahmadi , et al., “Agarose‐Based Biomaterials for Tissue Engineering,” Carbohydrate Polymers 187 (2018): 66–84, 10.1016/j.carbpol.2018.01.060. 29486846

[150] R. Sanz‐Horta , A. Matesanz , A. Gallardo , et al., “Technological Advances in Fibrin for Tissue Engineering,” Journal of Tissue Engineering 14 (2023): 20417314231190288, 10.1177/20417314231190288. 37588339 PMC10426312

[151] M. Ducret , A. Montembault , J. Josse , et al., “Design and Characterization of a Chitosan‐Enriched Fibrin Hydrogel for Human Dental Pulp Regeneration,” Dental Materials 35, no. 4 (2019): 523–533, 10.1016/j.dental.2019.01.018. 30712823

[152] Y. Huang , Q. Hu , X. Li , et al., “A Plasma‐Derived Fibrin *In Situ* Hydrogel for Sustained Release of Copper and Zinc Ions in Periodontitis Treatment,” Biomaterials Science 13, no. 23 (2025): 6662–6675, 10.1039/D5BM00951K. 41104966

[153] G. Li and S. Sun , “Silk Fibroin‐Based Biomaterials for Tissue Engineering Applications,” Molecules 27, no. 9 (2022): 2757, 10.3390/molecules27092757. 35566110 PMC9103528

[154] A. Vaziri , E. Vasheghani Farahani , and F. Bagheri , “Enzyme Crosslinked Injectable Silk Fibroin/Tragacanth Gum Bionanocomposite Hydrogels Embedding Bioactive Glass Nanoparticles With Potential for Bone Tissue Engineering,” Preprint *BioRxiv* (2025), 10.2139/ssrn.5709826.

[155] F. Mohammadzadeh , M. Gharivi , M. Hekmati , F. Montazeri , A. S. Mirmohammadali , and A. Malek Khachatourian , “Design and Characterization of Nitrogen‐Doped Reduced Graphene Oxide‐Enhanced Silk Fibroin/Gum Tragacanth Hydrogels for Wound Healing,” Results in Engineering 28 (2025): 107813, 10.1016/j.rineng.2025.107813.

[156] C. O. Crosby , B. Stern , N. Kalkunte , S. Pedahzur , S. Ramesh , and J. Zoldan , “Interpenetrating Polymer Network Hydrogels as Bioactive Scaffolds for Tissue Engineering,” Reviews in Chemical Engineering 38, no. 3 (2022): 347–361, 10.1515/revce-2020-0039. 35400772 PMC8993131

[157] T. Prebeg , Ž. Perić Kačarević , and G. Matijašić , “Hybrid Hydrogels for Bioink Development and Potential Use in Dental Tissue Engineering,” International Journal of Dental Biomaterials Research 1 (2023): 22–29, 10.56939/DBR23122p.

[158] A. Aldhaher , F. Shahabipour , A. Shaito , et al., “3D Hydrogel/Bioactive Glass Scaffolds in Bone Tissue Engineering: Status and Future Opportunities,” Heliyon 9, no. 7 (2023): e17050, 10.1016/j.heliyon.2023.e17050. 37483767 PMC10362084

[159] A. Sadeghianmaryan , S. Naghieh , Z. Yazdanpanah , et al., “Fabrication of Chitosan/Alginate/Hydroxyapatite Hybrid Scaffolds Using 3D Printing and Impregnating Techniques for Potential Cartilage Regeneration,” International Journal of Biological Macromolecules 204 (2022): 62–75, 10.1016/j.ijbiomac.2022.01.201. 35124017

[160] S. G , P. P T , A. Cecil , C. S , and N. Suresh , “Formulation of a Novel Polymeric Hydrogel Membrane for Periodontal Tissue Regeneration Using Tricalcium Phosphate‐Alginate Reinforcement,” Cureus 16 (2024): 57844, 10.7759/cureus.57844. PMC11078324 38721191

[161] Q. Min , J. Liu , X. Yu , Y. Zhang , J. Wu , and Y. Wan , “Sequential Delivery of Dual Growth Factors From Injectable Chitosan‐Based Composite Hydrogels,” Marine Drugs 17, no. 6 (2019): 365, 10.3390/md17060365. 31226756 PMC6627327

[162] M. Afzali , N. Esfandiaribayat , and J. Boateng , “Medicated and Multifunctional Composite Alginate‐Collagen‐Hyaluronate Based Scaffolds Prepared Using Two Different Crosslinking Approaches Show Potential for Healing of Chronic Wounds,” Drug Delivery and Translational Research 15, no. 7 (2025): 2483–2508, 10.1007/s13346-024-01745-0. 39661314 PMC12137399

[163] M. L. Gould , N. J. Downes , A. G. Woolley , et al., “Harnessing the Regenerative Potential of Purified Bovine Dental Pulp and Dentin Extracellular Matrices in a Chitosan/Alginate Hydrogel,” Macromolecular Bioscience 24, no. 11 (2024): 2400254, 10.1002/mabi.202400254. 38938070

[164] U. Shirbhate and P. Bajaj , “Injectable and Self‐Invigorating Hydrogel Applications in Dentistry and Periodontal Regeneration: A Literature Review,” Cureus 14 (2022): 29248, 10.7759/cureus.29248. PMC9578657 36277588

[165] M. S. Santos , A. B. Dos Santos , and M. S. Carvalho , “New Insights in Hydrogels for Periodontal Regeneration,” Journal of Functional Biomaterials 14, no. 11 (2023): 545, 10.3390/jfb14110545. 37998114 PMC10672517

[166] Q. Sun , Y. Li , P. Luo , and H. He , “Animal Models for Testing Biomaterials in Periodontal Regeneration,” Biomaterials Translational 4, no. 3 (2023): 142–150. 38283090 10.12336/biomatertransl.2023.03.003 PMC10817781

[167] S. Wu , Z. Chai , Y. Yang , et al., “Effect of Matrix Stiffness on the Osteogenic Differentiation of Human Periodontal Ligament Stem Cells in a Three‐Dimensional Culture Hydrogel: A Preliminary Study,” ACS Biomaterials Science & Engineering 11, no. 9 (2025): 5616–5626, 10.1021/acsbiomaterials.5c01151. 40856628

[168] M. Sheikh and A. Saiyyad , “Injectable Hydrogels for Cartilage and Bone Regeneration: Material Properties, Delivery Strategies, and Clinical Applications,” Medicine and Pharmacology 18 (2025): 70–81, 10.20944/preprints202501.1452.v2.

[169] M. Chatzinikolaidou , “Biofabrication of Complex Tissues for Bone Regeneration via Bioprinting,” Orthopaedic Proceedings 107‐B, no. SUPP_7 (2025): 85–85, 10.1302/1358-992X.2025.7.085.

[170] Z. Farimani , A. R. Shamshiri , H. Asl Roosta , S. Akbari , and M. Bohlouli , “Regenerative Benefits of Using Growth Factors in Treatment of Periodontal Defects: A Systematic Review and Meta‐Analysis With Trial Sequential Analysis on Preclinical Studies,” Journal of Tissue Engineering and Regenerative Medicine 15, no. 11 (2021): 964–997, 10.1002/term.3241. 34480421

[171] E. M. Ahmed , “Hydrogel: Preparation, Characterization, and Applications: A Review,” Journal of Advanced Research 6, no. 2 (2015): 105–121, 10.1016/j.jare.2013.07.006. 25750745 PMC4348459

[172] P. Sharma , S. Saurav , Z. Tabassum , et al., “Applications and Interventions of Polymers and Nanomaterials in Alveolar Bone Regeneration and Tooth Dentistry,” RSC Advances 14, no. 49 (2024): 36226–36245, 10.1039/D4RA06092J. 39534053 PMC11555558

[173] S. V. Gohil , A. Padmanabhan , H.‐M. Kan , M. Khanal , and L. S. Nair , “Degradation‐Dependent Protein Release From Enzyme Sensitive Injectable Glycol Chitosan Hydrogel,” Tissue Engineering Part A 27, no. 13–14 (2021): 867–880, 10.1089/ten.tea.2020.0124. 32940146 PMC8336245

[174] S. Taokaew , W. Kaewkong , and W. Kriangkrai , “Recent Development of Functional Chitosan‐Based Hydrogels for Pharmaceutical and Biomedical Applications,” Gels 9, no. 4 (2023): 277, 10.3390/gels9040277. 37102889 PMC10138304

[175] J. Li and D. J. Mooney , “Designing Hydrogels for Controlled Drug Delivery,” Nature Reviews Materials 1, no. 12 (2016): 16071, 10.1038/natrevmats.2016.71. PMC5898614 29657852

[176] M. S. Santos , J. C. Silva , and M. S. Carvalho , “Hierarchical Biomaterial Scaffolds for Periodontal Tissue Engineering: Recent Progress and Current Challenges,” International Journal of Molecular Sciences 25, no. 16 (2024): 8562, 10.3390/ijms25168562. 39201249 PMC11354458

[177] M. H. Norahan , S. Sivarasu , A. Fayzullin , et al., “Bioengineering of Periodontal Tissues: Cell Therapy and Biomaterials Application,” Bioengineering 12, no. 11 (2025): 1213, 10.3390/bioengineering12111213. 41301169 PMC12649470

[178] A. Ayala‐Ham , J. López‐Gutierrez , M. Bermúdez , et al., “Hydrogel‐Based Scaffolds in Oral Tissue Engineering,” Frontiers in Materials 8 (2021): 708945, 10.3389/fmats.2021.708945.

[179] I. Roato , B. Masante , G. Putame , D. Massai , and F. Mussano , “Challenges of Periodontal Tissue Engineering: Increasing Biomimicry Through 3D Printing and Controlled Dynamic Environment,” Nanomaterials 12, no. 21 (2022): 3878, 10.3390/nano12213878. 36364654 PMC9655809

[180] H. Wang , X. Chang , Q. Ma , et al., “Bioinspired Drug‐Delivery System Emulating the Natural Bone Healing Cascade for Diabetic Periodontal Bone Regeneration,” Bioactive Materials 21 (2023): 324–339, 10.1016/j.bioactmat.2022.08.029. 36185747 PMC9483739

[181] V. Kumar and A. K. Sundramoorthy , “Potential of Nature‐Derived Biopolymers for Oral Applications‐ A Mini‐Review,” Mini‐Reviews in Medicinal Chemistry 25, no. 7 (2025): 529–538, 10.2174/0113895575359305241218113847. 39757671

[182] M. Paczkowska‐Walendowska , M. Kulawik , J. Kwiatek , D. Bikiaris , and J. Cielecka‐Piontek , “Novel Applications of Natural Biomaterials in Dentistry—Properties, Uses, and Development Perspectives,” Materials 18, no. 9 (2025): 2124, 10.3390/ma18092124. 40363627 PMC12074186

[183] M. Gomez‐Florit , A. Pardo , R. M. A. Domingues , et al., “Natural‐Based Hydrogels for Tissue Engineering Applications,” Molecules 25, no. 24 (2020): 5858, 10.3390/molecules25245858. 33322369 PMC7763437

[184] N. Bu , L. Li , and X. Hu , “Recent Trends in Natural Polymer‐Based Hydrogels for Biomedical Applications,” Biofunctional Materials (2023): 4, 10.55092/bm20230009.

[185] H. Choi , W.‐S. Choi , and J.‐O. Jeong , “A Review of Advanced Hydrogel Applications for Tissue Engineering and Drug Delivery Systems as Biomaterials,” Gels 10, no. 11 (2024): 693, 10.3390/gels10110693. 39590049 PMC11594258

[186] B. Wang , F. Ge , W. Wang , B. Wang , C. J. Xian , and Y. Zhai , “Hydrogel‐Based Therapeutic Strategies for Periodontal Tissue Regeneration: Advances, Challenges, and Future Perspectives,” Pharmaceutics 17, no. 11 (2025): 1382, 10.3390/pharmaceutics17111382. 41304720 PMC12655002

[187] R. Dal‐Fabbro , A. Daghrery , C. Anselmi , et al., “Recent Advances in Injectable Hydrogel Biotherapeutics for Regenerative Dental Medicine,” Macromolecular Bioscience 25, no. 10 (2025): 00096, 10.1002/mabi.202500096. PMC12530706 40605036

[188] R. Chen , M. Zhu , C. Zhang , X. Xu , and J. Li , “Recent Advances in Natural Macromolecule‐Based Hydrogels for Treating Oral Soft Tissue Inflammation: A Review,” International Journal of Biological Macromolecules 340 (2026): 150234, 10.1016/j.ijbiomac.2026.150234. 41534810

[189] R. Gauthier , C. Jeannin , N. Attik , A.‐M. Trunfio‐Sfarghiu , K. Gritsch , and B. Grosgogeat , “Tissue Engineering for Periodontal Ligament Regeneration: Biomechanical Specifications,” Journal of Biomechanical Engineering 143, no. 3 (2020): 030801, 10.48550/ARXIV.2012.14683. 33067629

[190] Z. Gao , L. Hassouneh , X. Yang , J. Pang , P. D. Thornton , and G. Tronci , “Hydrogen Phosphate‐Mediated Acellular Biomineralisation Within a Dual Crosslinked Hyaluronic Acid Hydrogel,” European Polymer Journal 143 (2021): 110187, 10.48550/ARXIV.2101.02267.

[191] A. Poirier , P. L. Griel , T. Zinn , et al., “Energy Landscape of Sugar Conformation Controls the Sol‐to‐Gel Transition in Self‐Assembled Bola Glycolipid Hydrogels,” arXiv (2022): 2206.05106.

[192] İ. D. Külcü and R. Dargazany , “Mechanical Characterization of Stress Softening in Double Network Hydrogels,” arXiv (2017): 1712.07459.

[193] D. Kogan and M. Gottlieb , “SAOS and LAOS Rheology for Differentiating Chemical and Physical Crosslinking: A Case Study on PVA Hydrogels,” arXiv (2025): 2510.06720.

[194] P. L. Bourdonnec , C. Ferkous , L. Communal , L. Cipelletti , and R. Merindol , “Decoupling Dynamics and Crosslink Stability in Supramolecular Hydrogels Using Associative Exchange,” arXiv (2025): 2508.19937. 10.1002/adma.202516741 PMC13073121 41568874

[195] G. Tronci , H. Ajiro , S. J. Russell , D. J. Wood , and M. Akashi , “Tuneable Drug‐Loading Capability of Chitosan Hydrogels With Varied Network Architectures,” arXiv (2013): 1310.7461. 10.1016/j.actbio.2013.10.014 PMC4933205 24157693

[196] C. Seyrig , A. Poirier , P. Javier , and N. Baccile , “Interpenetrated Biosurfactant‐Biopolymer Orthogonal Hydrogels: The Biosurfactant's Phase Controls the Hydrogel's Mechanics,” arXiv (2022): 2212.10987. 10.1021/acs.biomac.2c00319 36576429

